# Supplementary material for: Implementation of an antibiotic resistance surveillance tool in Madagascar, the TSARA project: a prospective, observational, multicentre, hospital-based study protocol
Source: BMJ Open. 2024 Mar 19;14(3):e078504. doi: 10.1136/bmjopen-2023-078504 (PMC10953040; doi:10.1136/bmjopen-2023-078504)
Supplement: Supplementary data [file bmjopen-2023-078504supp001.pdf]

Supplementary material 1. TSARA microbiological questionnaire

Question	Name of the variable	Type of data	Format	Description
**Hospital where the sample was taken**	id_hosp	Single choice list	CHUJRB, CHUJRA, CHUMET, CHRR ANTSIRABE, CHU ANOSIALA, CHU MORAFENO , CHU PZAGA , CHU FIANARANTSOA, CHRR AMBOHIMIANDRA, CHU TULEAR	Hospital where the bacteriological sample was taken (hospital of inclusion)
**Patient identification number**	id_pat	Numeric	_ _ _ _ _ _ _ _ _ _	Patient's inclusion number
**Date of collection**	dt_pvt	Date	DD/MM/YYYY HH:MM	Date of collection
**Date of receipt of the sample in the laboratory**	dt_rec_pvt	Date	DD/MM/YYYY HH:MM	Date of receipt of the sample in the laboratory
**Date of bacteriological CR**	dt_cr_pvt	Date	DD/MM/YYYY HH:MM	Date of delivery of the result to the patient or prescribing physician
**Type of sampling**	pvt_type	Single choice list	Blood culture, Intravascular device, Ascites fluid, Cerebrospinal fluid, Joint fluid, Respiratory specimen, Deep tissue specimen, Genital specimen, Urine, Neonatal specimen and placenta, Superficial pus, Deep tissue specimen, Stool culture (not screening), Other specimen, Unknown	Type of sampling performed
**Positive culture**	culture	Single choice list	Yes/No	Identification of a microorganism on the culture
**If yes, identified pathogen**	bact	Single choice list	List of bacteria	Identification of the micro-organism on the sample
**Nalidixic acid**	nalidixic_acid	Single choice list	S, I, R, NOT TESTED	Sensitivity or resistance of the antibiotic tested
**Fusidic acid**	fusidic_acid	Single choice list	S, I, R, NOT TESTED	Sensitivity or resistance of the antibiotic tested
**Amikacin**	amikacin	Single choice list	S, I, R, NOT TESTED	Sensitivity or resistance of the antibiotic tested
**Amoxicillin**	amoxicillin	Single choice list	S, I, R, NOT TESTED	Sensitivity or resistance of the antibiotic tested
**Amoxicillin-clavulanic acid**	amoxicillin_clav	Single choice list	S, I, R, NOT TESTED	Sensitivity or resistance of the antibiotic tested
**Ampicillin**	ampicillin	Single choice list	S, I, R, NOT TESTED	Sensitivity or resistance of the antibiotic tested
**Ampicillin-Sulbactam**	ampicillin_sulbactam	Single choice list	S, I, R, NOT TESTED	Sensitivity or resistance of the antibiotic tested
**Azithromycin**	azithromycin	Single choice list	S, I, R, NOT TESTED	Sensitivity or resistance of the antibiotic tested
**Aztreonam**	aztreonam	Single choice list	S, I, R, NOT TESTED	Sensitivity or resistance of the antibiotic tested

**Cefadroxil**	cefadroxil	Single choice list	S, I, R, NOT TESTED	Sensitivity or resistance of the antibiotic tested
**Cefalexin**	cefalexin	Single choice list	S, I, R, NOT TESTED	Sensitivity or resistance of the antibiotic tested
**Cefalotin**	cefalotin	Single choice list	S, I, R, NOT TESTED	Sensitivity or resistance of the antibiotic tested
**Cefamandole**	cefamandole	Single choice list	S, I, R, NOT TESTED	Sensitivity or resistance of the antibiotic tested
**Cefazolin**	cefazolin	Single choice list	S, I, R, NOT TESTED	Sensitivity or resistance of the antibiotic tested
**Cefepime**	cefpime	Single choice list	S, I, R, NOT TESTED	Sensitivity or resistance of the antibiotic tested
**Cefixime**	cefixime	Single choice list	S, I, R, NOT TESTED	Sensitivity or resistance of the antibiotic tested
**Cefotaxime**	cefotaxime	Single choice list	S, I, R, NOT TESTED	Sensitivity or resistance of the antibiotic tested
**Cefoxitin**	cefoxitin	Single choice list	S, I, R, NOT TESTED	Sensitivity or resistance of the antibiotic tested
**Cefpodoxime**	cefpodoxime	Single choice list	S, I, R, NOT TESTED	Sensitivity or resistance of the antibiotic tested
**Ceftazidime**	ceftazidime	Single choice list	S, I, R, NOT TESTED	Sensitivity or resistance of the antibiotic tested
**Ceftriaxone**	ceftriaxone	Single choice list	S, I, R, NOT TESTED	Sensitivity or resistance of the antibiotic tested
**Cefuroxime**	cefuroxime	Single choice list	S, I, R, NOT TESTED	Sensitivity or resistance of the antibiotic tested
**Chloramphenicol**	chloramphenicol	Single choice list	S, I, R, NOT TESTED	Sensitivity or resistance of the antibiotic tested
**Chlortetracycline**	chlortetracycline	Single choice list	S, I, R, NOT TESTED	Sensitivity or resistance of the antibiotic tested
**Ciprofloxacin**	ciprofloxacin	Single choice list	S, I, R, NOT TESTED	Sensitivity or resistance of the antibiotic tested
**Clarithromycin**	clarithromycin	Single choice list	S, I, R, NOT TESTED	Sensitivity or resistance of the antibiotic tested
**Clindamycin**	clindamycin	Single choice list	S, I, R, NOT TESTED	Sensitivity or resistance of the antibiotic tested
**Cloxacillin**	cloxacillin	Single choice list	S, I, R, NOT TESTED	Sensitivity or resistance of the antibiotic tested
**Colistin**	colistin	Single choice list	S, I, R, NOT TESTED	Sensitivity or resistance of the antibiotic tested
**Daptomycin**	daptomycin	Single choice list	S, I, R, NOT TESTED	Sensitivity or resistance of the antibiotic tested
**Doxycycline**	doxycycline	Single choice list	S, I, R, NOT TESTED	Sensitivity or resistance of the antibiotic tested
**Ertapeneme**	ertapeneme	Single choice list	S, I, R, NOT TESTED	Sensitivity or resistance of the antibiotic tested
**Erythromycin**	erythromycin	Single choice list	S, I, R, NOT TESTED	Sensitivity or resistance of the antibiotic tested
**Fosfomycin**	fosfomycin	Single choice list	S, I, R, NOT TESTED	Sensitivity or resistance of the antibiotic tested
**Gentamicin**	gentamicin	Single choice list	S, I, R, NOT TESTED	Sensitivity or resistance of the antibiotic tested
**Imipeneme**	imipeneme	Single choice list	S, I, R, NOT TESTED	Sensitivity or resistance of the antibiotic tested
**Josamycin**	josamycin	Single choice list	S, I, R, NOT TESTED	Sensitivity or resistance of the antibiotic tested
**Levofloxacin**	levofloxacin	Single choice list	S, I, R, NOT TESTED	Sensitivity or resistance of the antibiotic tested

**Lincomycin**	lincomycin	Single choice list	S, I, R, NOT TESTED	Sensitivity or resistance of the antibiotic tested
**Linezolid**	linezolide	Single choice list	S, I, R, NOT TESTED	Sensitivity or resistance of the antibiotic tested
**Meropeneme**	meropeneme	Single choice list	S, I, R, NOT TESTED	Sensitivity or resistance of the antibiotic tested
**Metronidazole**	metronidazole	Single choice list	S, I, R, NOT TESTED	Sensitivity or resistance of the antibiotic tested
**Midecamycin**	midecamycin	Single choice list	S, I, R, NOT TESTED	Sensitivity or resistance of the antibiotic tested
**Minocycline**	minocycline	Single choice list	S, I, R, NOT TESTED	Sensitivity or resistance of the antibiotic tested
**Moxifloxacin**	moxifloxacin	Single choice list	S, I, R, NOT TESTED	Sensitivity or resistance of the antibiotic tested
**Neomycin**	neomycin	Single choice list	S, I, R, NOT TESTED	Sensitivity or resistance of the antibiotic tested
**Netilmycin**	netilmycin	Single choice list	S, I, R, NOT TESTED	Sensitivity or resistance of the antibiotic tested
**Nifuroxazide**	nifuroxazide	Single choice list	S, I, R, NOT TESTED	Sensitivity or resistance of the antibiotic tested
**Nitrofurantoin**	nitrofurantoin	Single choice list	S, I, R, NOT TESTED	Sensitivity or resistance of the antibiotic tested
**Norfloxacin**	norfloxacin	Single choice list	S, I, R, NOT TESTED	Sensitivity or resistance of the antibiotic tested
**Ofloxacin**	ofloxacin	Single choice list	S, I, R, NOT TESTED	Sensitivity or resistance of the antibiotic tested
**Oxacillin**	oxacillin	Single choice list	S, I, R, NOT TESTED	Sensitivity or resistance of the antibiotic tested
**Pefloxacin**	pefloxacin	Single choice list	S, I, R, NOT TESTED	Sensitivity or resistance of the antibiotic tested
**Piperacillin**	piperacillin	Single choice list	S, I, R, NOT TESTED	Sensitivity or resistance of the antibiotic tested
**Piperacillin-Tazobactam**	piperacillin_tazo	Single choice list	S, I, R, NOT TESTED	Sensitivity or resistance of the antibiotic tested
**Pivmecillinam**	pivmecillinam	Single choice list	S, I, R, NOT TESTED	Sensitivity or resistance of the antibiotic tested
**Polymyxin**	polymyxin	Single choice list	S, I, R, NOT TESTED	Sensitivity or resistance of the antibiotic tested
**Pristinamycin**	pristinamycin	Single choice list	S, I, R, NOT TESTED	Sensitivity or resistance of the antibiotic tested
**Rifampicin**	rifampin	Single choice list	S, I, R, NOT TESTED	Sensitivity or resistance of the antibiotic tested
**Spectinomycin**	spectinomycin	Single choice list	S, I, R, NOT TESTED	Sensitivity or resistance of the antibiotic tested
**Streptomycin**	streptomycin	Single choice list	S, I, R, NOT TESTED	Sensitivity or resistance of the antibiotic tested
**Sulfamethoxazole**	sulfamethoxazole	Single choice list	S, I, R, NOT TESTED	Sensitivity or resistance of the antibiotic tested
**Tedizolide**	tedizolide	Single choice list	S, I, R, NOT TESTED	Sensitivity or resistance of the antibiotic tested
**Teicoplanin**	teicoplanin	Single choice list	S, I, R, NOT TESTED	Sensitivity or resistance of the antibiotic tested
**Telithromycin**	telithromycin	Single choice list	S, I, R, NOT TESTED	Sensitivity or resistance of the antibiotic tested
**Temocillin**	temocillin	Single choice list	S, I, R, NOT TESTED	Sensitivity or resistance of the antibiotic tested
**Ticarcillin**	ticarcillin	Single choice list	S, I, R, NOT TESTED	Sensitivity or resistance of the antibiotic tested

**Ticarcillin-clavulanic acid**	ticarcillin_clav	Single choice list	S, I, R, NOT TESTED	Sensitivity or resistance of the antibiotic tested
**Tigecycline**	tigecycline	Single choice list	S, I, R, NOT TESTED	Sensitivity or resistance of the antibiotic tested
**Tobramycin**	tobramycin	Single choice list	S, I, R, NOT TESTED	Sensitivity or resistance of the antibiotic tested
**Vancomycin**	vancomycin	Single choice list	S, I, R, NOT TESTED	Sensitivity or resistance of the antibiotic tested

Supplementary material 2. TSARA Clinical Questionnaire

Question	Name of the variable	Type of data	Format	Description
**Date of visit in the room**	dt_visit	Date	DD/MM/YYYY	Date of patient/clinician visit to clinical service
**Gender**	gender	Unique choice	Male, Female	Sex of the patient
**Date of birth**	dt_naiss	Date	DD/MM/YYYY	Patient's date of birth
**Age**	age	Numeric	_ _ _	Age of patient at hospital admission in years
**Size**	size	Numeric	_ _ _	Patient height (cm) at admission
**Weight**	weight	Numeric	_ _ _	Patient weight (kg) at admission
**Status at birth**	status_naiss	Single choice list	Premature, Born at term	Premature status at the birth of the child
**Patient's residence**	place_resid	Single choice list	Urban, Rural	Patient's primary place of residence on admission
**Patient's region of residence**	reg_resid	Text	_ _ _ _ _ _ _ _ _ _ _ _ _ _ _	Patient's primary area of residence on admission
**Date of admission to hospital**	dt_adm_hosp	Date	DD/MM/YYYY	Date of hospital entry (TSARA participant)
**Patient's origin**	from	Single choice list	Home, Transfer to anotherhospital, Unknown	Patient's origin before admission to hospital
**Diagnosis at admission**	diag_adm	Single choice list	Infectious, Non-infectious, Unknown	Patient diagnosis on admission to hospital
**Type of service**	type_service	Single choice list	Medicine, Surgery, Intensive Care, Gyneco-Obstetrics,Pediatrics, Other	Type of service where the sample was taken
**Respiratory rate (beats per minute)**	freq_resp	Numeric	_ _ _	Patient's respiratory rate on admission
**Heart rate (beats per minute)**	freq_card	Numeric	_ _ _	Patient's heart rate on admission
**Systolic blood pressure (mmHg)**	pa_sys	Numeric	_ _ _	Systolic blood pressure on admission
**Disorder of consciousness**	awareness	Single choice list	Yes/No	Presence of disturbed consciousness at admission
**Peripheral venous access**	vvp	Single choice list	Yes/No	Placement of a PVV between admission and collection
**Central venous access**	vvc	Single choice list	Yes/No	Placement of a CVC between admission and

				collection
**Urinary catheterization**	probe_uro	Single choice list	Yes/No	Placement of a urinary catheter between admission andsampling
**Oro-tracheal intubation**	intubation	Single choice list	Yes/No	Intubation performed between admission and collection
**Reason for collection**	motif_pvt	Multiple choice list	List of reason for samples	Reason for collection
**Date of onset of symptoms**	dt_sympt	Date	DD/MM/YYYY	Date of onset of the first symptoms for which the sample was taken
**Antibiotic history within 3 months prior to admission**	atcd_atb	Single choice list	Yes/No	Use of ATB within 3 months of hospital admission
**If yes, specify ATBs**	type_atcd_atb	Multiple choice list	List of antibiotics	If yes, specify the type of ATB
**Antibiotic therapy during the stay before sampling**	atb_hosp	Single choice list	Yes/No	Antibiotic treatment prescribed between admission to hospital and sampling
**If yes, specify ATBs**	type_atb_hosp	Multiple choice list	List of antibiotics	If yes, specify the type of ATB
**Antibiotic therapy after receiving the antibiogram**	atb_post_atbg	Single choice list	Yes/No	Antibiotic treatment prescribed after receiving the antibiogram
**If yes, specify ATBs**	type_atb_post_atbg	Multiple choice list	List of antibiotics	If yes, specify the type of ATB
**History of hospitalization in the 6 months preceding thehospitalization**	atcd_hosp	Single choice list	Yes/No	History of hospitalization in the 6 months preceding thehospitalization
**History of surgery in the 6 months preceding hospitalization**	atcd_chir	Single choice list	Yes/No	History of surgery in the 6 months preceding hospitalization
**Comorbidities**	comorbidities	Multiple choice list	List of comorbidities	Presence of clinical comorbidities at admission
**Other comorbidities**	other_comorbidity	Free text		Other comorbidities

Supplementary material 3. TSARA III follow-up questionnaire

Question	Name of the variable	Type of data	Format	Description
**Date of discharge from hospital**	dt_end_hosp	Date	DD/MM/YYYY	End date of hospital stay or date of death if the patient has deceased.
**Exit diagnosis**	diag_hosp	Single choice list	List of diagnosis	
**Patient status at discharge**	hosp_output	Single choice list	Return home, Deceased, Transfer to another hospital	Patient status at the end of the hospital stay

Supplementary material 4. Description of facilities included in the TSARA project

	CHU[a] Joseph Ravoahangy Andrianavalona (JRA)	CHU Joseph Raseta Befalatanana (JRB)	CHU Mère-Enfant Tsaralalana (MET)	CHU Ambohimiandra	CHU Anosiala	CHRR[b] Antsirabe	CHU Andrianjato	CHU Pzaga	CHU Morafeno	CHU Toliary
**City**	Antananarivo	Antananarivo	Antananarivo	Antananarivo	Anosiala	Antsirabe	Fianarantsoa	Mahajanga	Toamasina	Toliary
**Type of hospital[c]**	Tertiary	Tertiary	Tertiary	Tertiary	Tertiary	Secondary	Tertiary	Tertiary	Tertiary	Tertiary
**University/Regional hospital**	Teaching	Teaching	Teaching	Teaching	Teaching	Regional	Teaching	Teaching	Teaching	Teaching
**Hospital size (number of beds)**	595 beds	450 beds	200 beds	44 beds	180 beds	70 beds	120 beds	303 beds	120 beds	40 beds
**Hospital ownership**	Public	Public	Public	Public	Public	Public	Public	Public	Public	Public
**Type of population**	Adult	Adult	Pediatrics	Adult & Pediatrics	Adult	Adult	Adult	Adult	Adult	Adult
**Presence of ICU[d]**	Yes	Yes	Yes	Yes	Yes	Yes	Yes	Yes	Yes	Yes
**Strengthened bacteriology laboratory**	Yes	Yes	Yes	Yes	Yes	Yes	Yes	Yes	Yes	Yes
**Diagnostic AST[e] method**	Disk diffusion (EUCAST v. 2021)	Disk diffusion (EUCAST v. 2021)	Disk diffusion (EUCAST v. 2021)	Disk diffusion (EUCAST v. 2021)	Disk diffusion (EUCAST v. 2021)	Disk diffusion (EUCAST v. 2021)	Disk diffusion (EUCAST v. 2021)	Disk diffusion (EUCAST v. 2021)	Disk diffusion (EUCAST v. 2021)	Disk diffusion (EUCAST v. 2021)
**Local guidelines on the use of antibiotics in place**	None	None	None	None	None	None	None	None	None	None

[a]CHU : Centre hospitalier Universitaire (Teaching hospital)
[b]CHRR : Centre Hospitalier de Référence régionale (Regional hospital)
[c]https://www.who.int/publications/i/item/WHO-EMP-IAU-2018.01
[d]ICU: Intensive Care Unit
[e]AST: Antibiotic susceptibility testing
