# Supplementary material for: Implementation of an antibiotic resistance surveillance tool in Madagascar, the TSARA project: a prospective, observational, multicentre, hospital-based study protocol
Source: BMJ Open. 2024 Mar 19;14(3):e078504. doi: 10.1136/bmjopen-2023-078504 (PMC10953040; doi:10.1136/bmjopen-2023-078504)
Supplement: Author's manuscript [file bmjopen-2023-078504.draft_revisions.pdf]

BMJ Open is committed to open peer review. As part of this commitment we make the peer review history of every article we publish publicly available.

When an article is published we post the peer reviewers' comments and the authors' responses online. We also post the versions of the paper that were used during peer review. These are the versions that the peer review comments apply to.

The versions of the paper that follow are the versions that were submitted during the peer review process. They are not the versions of record or the final published versions. They should not be cited or distributed as the published version of this manuscript.

BMJ Open is an open access journal and the full, final, typeset and author-corrected version of record of the manuscript is available on our site with no access controls, subscription charges or pay-per-view fees (http://bmjopen.bmj.com).

If you have any questions on BMJ Open's open peer review process please email info.bmjopen@bmj.com

BMJ Open

Implementation of an antimicrobial stewardship program in Madagascar, the TSARA project: a prospective, multicenter, hospital-based study protocol.

Journal:	*BMJ Open*
Manuscript ID	bmjopen-2023-078504
Article Type:	Protocol
Date Submitted by the Author:	03-Aug-2023
Complete List of Authors:	Elias, Christelle; Service Hygiène, Epidémiologie et Prévention, Hospices Civils de Lyon , ; Public Health, Epidemiology & Evolutionary Ecology of Infectious Diseases (PHE3ID), Centre International de Recherche en Infectiologie (CIRI), Inserm U1111, CNRS UMR5308, ENS de Lyon, Université Claude Bernard Lyon 1,<br>Raad, Mathieu; Fondation Mérieux, Direction des Opérations Internationales<br>Rasoanandrasana, Saida; Hôpital Befelatanana, Service de Biologie<br>Raherinandrasana, Antso Hasina; Faculté de Médecine de l'Université d'Antananarivo, Département de Santé Publique; Gouvernement de Madagascar Ministère de la Santé Publique, Direction des Hôpitaux de Régions et de Régions<br>Andriananja, Volatiana; Hôpital Befelatanana, Service des Maladies Infectieuses<br>Raberahona, Mihaja; University Hospital Joseph Raseta Befelatanana, Infectious Diseases<br>Moore, Catrin; St. George's, University of London, Centre for Neonatal and Paediatric Infection<br>Randria, Mamy; Hôpital Befelatanana, Service de Biologie<br>Raskine, Laurent; Fondation Mérieux, Direction des Opérations Internationales<br>Vanhems, Philippe; Université de Lyon; Université Lyon 1; CNRS UMR 5558, Biométrie et Biologie Evolutive, Epidémiologie et Santé Publique, Lyon, France, Hospices Civils de Lyon, Hôpital Edouard Herriot, Service d'Hygiène, Epidémiologie et Prévention, Lyon, France<br>Babin, François-Xavier; Fondation Mérieux, Direction des Opérations Internationales
Keywords:	BACTERIOLOGY, Protocols & guidelines < HEALTH SERVICES ADMINISTRATION & MANAGEMENT, Diagnostic microbiology < INFECTIOUS DISEASES, Epidemiology < INFECTIOUS DISEASES

SCHOLARONE™
Manuscripts

Implementation of an antimicrobial stewardship program in Madagascar, the TSARA project: a prospective, multicenter, hospital-based study protocol.

C.Elias[1,2], M. Raad[3], S. Rasoanandrasana[4], AH. Raherinandrasana[5,6], V. Andriananja[7], M. Raberahona[7], C.E. Moore[8], M. Randria[4], L. Raskine[3], P Vanhems[1,2], FX. Babin[3]

1. Service Hygiène et Epidémiologie, Hospices Civils de Lyon, Lyon, France
2. Public Health, Epidemiology & Evolutionary Ecology of Infectious Diseases (PHE3ID) team, Centre International de Recherche en Infectiologie (CIRI), Inserm U1111, CNRS UMR5308, ENS de Lyon, Université Claude Bernard Lyon 1, Lyon, France
3. Direction des Opérations Internationales, Fondation Mérieux, Lyon, France
4. Service de Biologie, Hôpital Befelatanana, Antananarivo, Madagascar
5. Département de Santé Publique, Faculté de Médecine de l'Université d'Antananarivo, Madagascar
6. Direction des Hôpitaux de Régions et de Régions, Ministère de la Santé Publique de Madagascar
7. Service des Maladies Infectieuses, Hôpital Befelatanana, Antananarivo, Madagascar
8. Centre for Neonatal and Paediatric Infection, St. George's, University of London, London

Corresponding Author:

Dr Christelle ELIAS

Service Hygiène et Epidémiologie, Hospices Civils de Lyon, Lyon

165 Chemin du Grand Revoyet, 69645 PIERRE-BENITE Cedex

christelle.elias@chu-lyon.fr

Phone: +33 4 78 86 12 73

Keywords: Antimicrobial Resistance, AMR, Antimicrobial Stewardship, Developing countries, Guidelines, Hospital, Low- and- middle-income countries, Madagascar, Surveillance

For peer review only - http://bmjopen.bmj.com/site/about/guidelines.xhtml

Abstract

Introduction: Antimicrobial resistance (AMR) has become a significant public health threat. Without any interventions, AMR will account for 10 million deaths annually by 2050, this mainly affects low- and- middle-income countries. AMR has a systemic negative perspective: from the overall healthcare system to affecting the patient's personal outcome. In response to this issue, the WHO urged countries to provide antimicrobial stewardship programs (ASP). ASP in hospitals are a vital component of national action plans on AMR, and have been shown to significantly reduce AMR, in particular in low-income countries such as Madagascar.

As part of an ASP, AMR surveillance provides essential information needed to guide medical practice. We developed an AMR surveillance tool - TSARA - with the support of the Mérieux Foundation. TSARA combines bacteriological and clinical information to provide a better understanding of the scope and the effects of AMR in Madagascar, where no such surveillance tool is in place.

Methods and analysis: A prospective, hospital-based study was carried out for data collection using a standardized data collection tool, called TSARA deployed in 2023 in 10 hospitals in Madagascar participating to the national Malagasy laboratory network (RESAMAD). Any hospitalized patient where the clinician decided to take a bacterial sample is included. As a prospective study, individual isolate-level data and antimicrobial susceptibility information on pathogens were collected routinely from the bacteriology laboratory and compiled with clinical information retrieved from face-to-face interviews with the patient and completed by medical records where necessary. Analysis of the local ecology, resistance rates and antibiotic prescription patterns will be collected.

Ethics and dissemination: This protocol obtained ethical approval was obtained from the Malagasy Ethical Committee n°07-MSANP/SG/AGMED/CNPV/CERBM on January 24, 2023. Findings generated will be shared to national health stakeholders, microbiologists, members of the RESAMAD Network and the Malagasy academic society of Infectious diseases.

For peer review only

Strengths and limitations of this study

- This study provides an innovative and comprehensive approach in the field of AMR surveillance combining microbiological, clinical and prescription data to optimize the use and prescription of antibiotics.
- TSARA is a challenging study regarding the implementation of an antimicrobial stewardship program in a low-resource setting such as Madagascar.
- The TSARA project is highly dependent on the infrastructure and the available resources in Malagasy laboratories.

For peer review only

For peer review only - http://bmjopen.bmj.com/site/about/guidelines.xhtml

Introduction

Antimicrobial resistance (AMR) is a global health threat associated with a poor prognosis and an increased mortality in the population infected with resistant bacteria. In the coming decades, deaths attributable to AMR will overcome the mortality due to non-communicable diseases (1). Recent data from the Global Burden of Disease (2) suggested that the impact of AMR on health is even more important than previous estimations (1). To address the increasing problem, the World Health Organization (WHO) published the Global Action Plan on AMR in 2015 (3) and urged countries to build national action plans which aimed to plan interventions to combat AMR rapidly. One of the objectives was to implement and strengthen surveillance systems to monitor AMR as a first step of an antimicrobial stewardship program (ASP) (4), in particular in hospital settings where high levels of resistance occur. In addition to these measures, the WHO also advocates for the implementation of antibiotic management policies adapted to local resistance patterns in hospitals (5–8). Setting up an ASP is challenging, especially in low- and- middle-income countries (LMICs) due to the lack of infrastructure and governance (7,9). For a country like Madagascar, where decent healthcare facilities, infrastructures and laboratories are very scarce, collecting resistance data remains a challenge.

Madagascar is the fifth largest island in the world, but is considered a low-income country according to the World Bank (10). Health coverage in Madagascar is extremely limited and the patient has to directly afford the majority of health care (i.e. diagnostics, treatment, consumables, surgery). The majority of drugs - especially antibiotics - is dispensed only with a medical prescription. However, in Madagascar, it is very common to deliver such medicines “Over The Counter” and antibiotics can be sold easily, for those who can afford them, without any medical prescription. Lack or substandard infection control practices contribute significantly to the spread of bacterial resistance, both in the community (wastewater problem) and in hospitals (hospital hygiene and nosocomial transmission). The culture of systematic treatment against an infectious hypothesis encourages the misuse as well as the overconsumption of antibiotics (prescribed or not). At the hospital level, AMR has a greater effect, as patients are in hospital for several days, receive more care, have invasive procedures performed and thus are more at risk of healthcare-associated infections. Appropriate hygiene measures and standard precautions are difficult to implement in low-resources settings, where there is for instance an average of five hospital beds per room in a Malagasy university hospital.

For peer review only - http://bmjopen.bmj.com/site/about/guidelines.xhtml

Very few laboratories are able to perform cultures and antimicrobial susceptibility testing (AST) in Madagascar and the cost remains prohibitive for many patients. Targeted antibiotic therapy is therefore very little prescribed and antibacterial treatment often remains empirical without any de-escalation. In this context of low access to bacteriological laboratory combined with high rates of bacterial resistance at the national level, practitioners use broad-spectrum therapies, encouraging the selection pressure on the bacteria and creating emerging resistances. Knowledge of the local resistance patterns is essential to adapt the clinical decision-making according to locally adapted recommendations (8). Table 1 provides a SWOT (Strengths, Weaknesses, Opportunities and Threats) analysis as an overview of what is currently in place and the starting points of the implementation of an ASP in Madagascar.

For peer review only

Table 1. SWOT analysis of the implementation of an ASP in hospitals in Madagascar

**Strengths**<br>– Laboratory network RESAMAD (11) currently in place, already transferring data to the WHO through the GLASS-AMR module<br>– Active support of international stakeholders<br>– Identification of highly motivated Malagasy practitioners willing to work on AMR and ASP<br>– Existence of several scientific societies (infectious diseases, pediatrics, medical biology[1])	**Weaknesses**<br>– Little knowledge on the local epidemiology of AMR<br>– Attainment of antibiograms not performed on a daily basis<br>– Lack of visibility on antibiotic prescription in hospitals and its efficiency<br>– Implementation only doable where good laboratories exist<br>– Bacteriology results sometimes produced in a paper format
**Opportunities**<br>– Capture high level antibiotic use in hospitals<br>– Existing antibiotic recommendations in place, but they are not adapted to local resistance patterns (12)<br>– Existing AMR protocols including the surveillance of antimicrobial use	**Threats**<br>– Challenging implementation<br>– Substantial missing data<br>– Training personnel<br>– Funding<br>– Political instability<br>– Climate disorders

[1] Société de Pathologie Infectieuse de Madagascar, Société Malgache de Pédiatrie, SOMABIO

For peer review only - http://bmjopen.bmj.com/site/about/guidelines.xhtml

In Madagascar as well as in multiple developing countries, the Mérieux Foundation has been strengthening clinical laboratories in the fight against infectious diseases for decades to enhance the quality and access to clinical laboratories aiming at preventing infections and improving patient care (13). The Mérieux Foundation has been investing on the fight against AMR by supporting countries by developing infrastructures, laboratories, personnel training as well as building a laboratory network to strengthen laboratory-based surveillance of diseases. As a result, in 2022, 14 Malagasy laboratories performed bacteriology diagnosis among the RESAMAD laboratory network, which was created in 2007 and includes 27 laboratories across the country to date (RESAMAD - Fondation Mérieux (fondation-merieux.org)).

Given this context, and the challenge to combat AMR in healthcare settings, ASP in hospitals are a vital component of national action plans for AMR, and have been shown to significantly reduce AMR, in particular in low-income country such as Madagascar (9,14). As part of an ASP, AMR surveillance provides essential information needed to guide medical practice. An AMR surveillance tool called TSARA (Technique de Surveillance Actualisée de la Résistance aux Antimicrobiens) was developed thanks to the support of the Mérieux Foundation, with the objective of combining patient data with laboratory and epidemiological surveillance data to provide a better understanding of the scope and the effects of AMR in Madagascar, where no such surveillance tool is in place, in order to treat patients more effectively. It will allow monitoring and follow-up compared to the few existing prescription data (15). The TSARA project also aligns with the antimicrobial surveillance program led by the Direction of epidemiological surveillance and Response of the Ministry of Health in the context of One Health and in collaboration with other Ministries to tackle AMR in Madagascar. TSARA also answers one of the objective of the Malagasy national action plan on AMR.

For peer review only

Objectives

One of the most important benefits of AMR surveillance at the pathogen level is to achieve a standardized, comparable, and easily validated data collection method for quality data that is used to implement better treatment and guidelines (16). The data allows an optimization of the patient care by adapting treatment guidelines according the patient demographics and type of infection, with consideration of the circulating pathogens and local resistance patterns. The primary objective of TSARA is:

1. To institute a systematic approach towards microbiological data and clinical data collection and analysis of antimicrobial susceptibility information to optimize antimicrobial therapy in Malagasy hospitals.

The secondary objectives of TSARA are:

1. To establish timely reporting of information on pathogens of interest and their antimicrobial susceptibility profiles;
2. To allow monitoring of the hospital antibiotic prescription (quantity, quality and spectrum, adequacy with bacteriological results);
3. To provide data to the laboratory quality assessment and identify areas for improvement.

For peer review only

For peer review only - http://bmjopen.bmj.com/site/about/guidelines.xhtml

Methods and analysis

Study design and setting

Study design

TSARA is a prospective, multicenter, hospital-based study based on a standardized data collection tool deployed in ten facilities with both adult and pediatric patients in Madagascar (Figure 1), collecting microbiological, demographic, prescription and clinical data in one place.

Figure 1. Map of the laboratories and implementation sites of TSARA in Madagascar

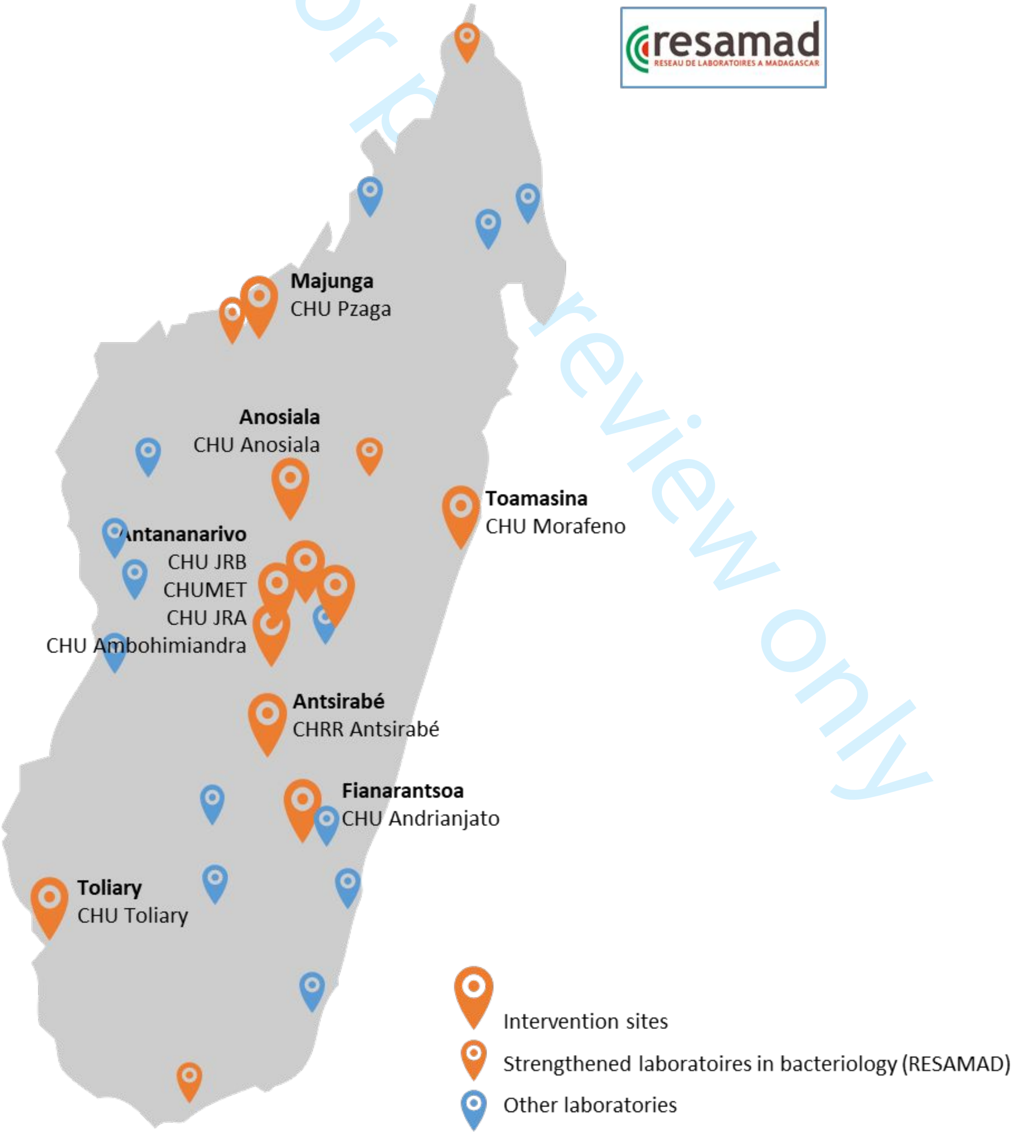

For peer review only - http://bmjopen.bmj.com/site/about/guidelines.xhtml

Outcome measures

The outcomes of interest will be categorized into behavioural, clinical and microbiological outcomes. Behavioural outcomes include changes in prescribing practices among prescribers and compliance to AMS protocols. A metric will be used to describe changes in prescribing practices e.g. number of prescriptions adapted to the results of the bacterial culture.

Clinical outcomes include mortality or length of stay in hospital. Microbiological outcomes include a description of the bacteria isolated by culture, bacterial resistance rates results as well as the delay between the sample being collected and the laboratory results communicated to the prescriber.

Setting and laboratory capacity

Table 2 describes the infrastructures in each hospital included in the TSARA project.

For peer review only

For peer review only - http://bmjopen.bmj.com/site/about/guidelines.xhtml

Table 2. Description of facilities included in the TSARA project

	CHU[a] Joseph Ravoahangy Andrianavalona (JRA)	CHU Joseph Raseta Befalatanana (JRB)	CHU Mère-Enfant Tsaralalana (MET)	CHU Ambohimiandra	CHU Anosiala	CHRR[b] Antsirabe	CHU Andrianjato	CHU Pzaga	CHU Morafeno	CHU Toliary
**City**	Antananarivo	Antananarivo	Antananarivo	Antananarivo	Anosiala	Antsirabe	Fianarantsoa	Mahajanga	Toamasina	Toliary
**Type of hospital[c]**	Tertiary	Tertiary	Tertiary	Tertiary	Tertiary	Secondary	Tertiary	Tertiary	Tertiary	Tertiary
**University/Regional hospital**	Teaching	Teaching	Teaching	Teaching	Teaching	Regional	Teaching	Teaching	Teaching	Teaching
**Hospital size (number of beds)**	595 beds	450 beds	200 beds	44 beds	180 beds	70 beds	120 beds	303 beds	120 beds	40 beds
**Hospital ownership**	Public	Public	Public	Public	Public	Public	Public	Public	Public	Public
**Type of population**	Adult	Adult	Pediatrics	Adult & Pediatrics	Adult	Adult	Adult	Adult	Adult	Adult
**Presence of ICU[d]**	Yes	Yes	Yes	Yes	Yes	Yes	Yes	Yes	Yes	Yes
**Strengthened bacteriology laboratory**	Yes	Yes	Yes	Yes	Yes	Yes	Yes	Yes	Yes	Yes
**Diagnostic AST[e] method**	Disk diffusion (EUCAST v. 2021)	Disk diffusion (EUCAST v. 2021)	Disk diffusion (EUCAST v. 2021)	Disk diffusion (EUCAST v. 2021)	Disk diffusion (EUCAST v. 2021)	Disk diffusion (EUCAST v. 2021)	Disk diffusion (EUCAST v. 2021)	Disk diffusion (EUCAST v. 2021)	Disk diffusion (EUCAST v. 2021)	Disk diffusion (EUCAST v. 2021)
**Local guidelines on the use of antibiotics in place**	None	None	None	None	None	None	None	None	None	None

[a]CHU : Centre hospitalier Universitaire (Teaching hospital)
[b]CHRR : Centre Hospitalier de Référence régionale (Regional hospital)
[c]https://www.who.int/publications/i/item/WHO-EMP-IAU-2018.01
[d]ICU: Intensive Care Unit
[e]AST: Antibiotic susceptibility testing

For peer review only - http://bmjopen.bmj.com/site/about/guidelines.xhtml

All laboratories participating in the TSARA project are part of the nationwide RESAMAD network in Madagascar (11). These laboratories perform diagnostics and antimicrobial susceptibility testing for infectious diseases, and have the capacity to perform phenotypic confirmation of the presence of resistance. To participate in TSARA, laboratories have to meet minimal quality standards requirements defined by the RESAMAD network and participate in an external quality assessment program. The laboratories also have the required infrastructure, equipment, supplies and resources to perform AST following EUCAST standards. The reporting of results to clinicians are made by qualified and trained technicians supervised by clinical microbiologists. The Mérieux Foundation performs regular training to laboratory staff and support the purchase of consumables (i.e. Petri dish, antibiotic disks) and reagents.

Study population

The population of the study includes all patients arriving at the hospital who are inpatients and have bacterial samples taken by the physician.

Inclusion criteria

All patients arriving at each hospital, who have a bacterial culture performed are included in the study. All positive and negative bacteriological samples from hospitalized patients in one of the participating institutions from January 2023 onwards will be included.

Exclusion criteria

The following criteria will be excluded from the TSARA project:

- All non-bacteriological specimens (virological, fungal, parasitic)
- All samples from patients not hospitalized >24 hours (emergency, consultation, outpatient hospitalization)
- All environmental samples (water, air, surfaces, medical devices)
- All post-mortem samples
- All samples reported as contaminated
- All specimens for which culture was not possible
- All specimens with laboratory non-compliance
- All the patients or legal guardians who object to be included in the TSARA project

For peer review only - http://bmjopen.bmj.com/site/about/guidelines.xhtml

For peer review only

- All patients with severe cognitive impairment
- All patients who do not have a full understanding of the French or Malagasy language

Participant timeline

Enrolled patients will be followed up during their hospital stay until discharge or death. Information on the vital status (alive, deceased, discharged or transferred to another hospital) will be collected.

Patient and public involvement

Patients and the public were not involved in the design of this study.

Data collection

Figure 2 describes the TSARA methodology. Individual isolate-level data and AST on pathogens were collected routinely from the bacteriology laboratory using a first questionnaire (Supplementary material 1). These data were compiled using a second questionnaire with basic clinical, demographic, and epidemiological information (including current antibiotic prescription) retrieved daily through face-to-face interviews with the patient and completed by medical records where necessary (Supplementary material 2). A third questionnaire was then filled in regarding follow-up data (Supplementary material 3). The data retrieved from TSARA meet the criteria and are linked to the GRAM project that seeks to estimate the burden of AMR worldwide (16, 17).

Figure 2. Methodology of the TSARA project

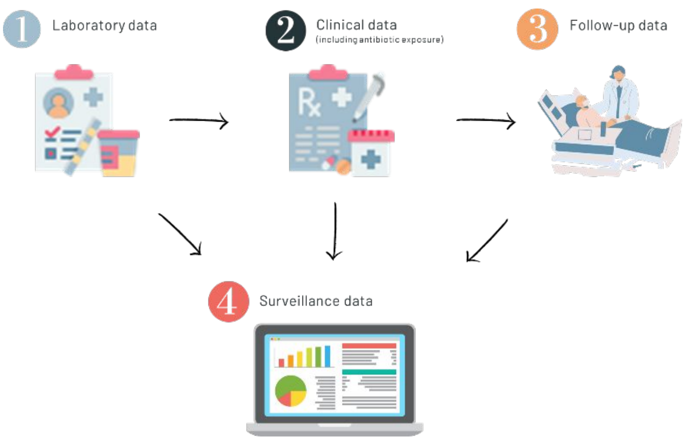

For peer review only - http://bmjopen.bmj.com/site/about/guidelines.xhtml

1. Collection of microbiological data
2. Collection of clinical data (including antibiotic prescription data)
3. Collection of follow-up data
4. Compilation of microbiological, clinical and follow-up data

To enhance data collection and limit data entry bias, data were directly captured on a tablet connected to the Epicollect5® platform (https://five.epicollect.net). Data were collected by Malagasy investigators (medical interns, biologists or clinical research associates) using tablets dedicated to this project, who received specific training on the TSARA methodology prior to the start of the study. The investigator consults the bacteriological samples daily, or as frequently as possible. A new eligible sample corresponds to a new inclusion in the TSARA project.

Statistical methods

Sample size

TSARA is a surveillance project, as such there is no calculation on the number of subjects needed *a priori*. Statistical analyses will be adapted *a posteriori* according to the total number of inclusions during the project period and the power needed to obtain valid results.

Description of the statistical methods

Any anomalies in the data will be checked with medical records during data cleaning. Data will be validated by quality control to identify outliers and inconsistencies by means of consistency tests (e.g. filling in the inclusion criteria, date of admission to hospital later than the date of admission to the department, date of death later than the date of onset of symptoms).

Descriptive summary tables with numbers and frequencies will be drawn up. Similarly, visual representations of the variables may be made to detect irregular or missing values. Any changes to the data will be documented and recorded separately from the raw database. Recoded variables will also be filled in (e.g. age categories).

A flow chart will be developed to describe the number of eligible, included and excluded patients and samples in our study. A descriptive analysis of the entire baseline population will be undertaken to identify the baseline characteristics of the study population (the specimen, bacterial, clinical and patient levels).

For peer review only - http://bmjopen.bmj.com/site/about/guidelines.xhtml

Categorical variables will be reported as frequencies and percentages of the total population. Quantitative variables will be described using the mean and standard deviation if the distribution is normal, or using the median and interquartile range if the distribution does not follow the normal distribution. The range with the minimum and maximum of the values will be filled in. For each variable, the proportion of missing values will be indicated.

Stratified analyses will also be provided to present results by facility, department, type of sample, and bacteria of interest (including species, and antibiotic resistance of interest) as well as by type of antibiotics prescribed.

Univariate and then multivariate analysis by a logistic regression will be performed to estimate the associations between the presence of resistance and the other variables. The p-value of each test will be reported. Only the variables with a p-value lower than 0.2 will be retained to perform the multivariate analyses. In addition to the presentation of the multivariate analyses, the most efficient multivariate model will be presented after a top-down selection of only those variables with a p-value less than 0.05. Stratified analyses will be conducted to identify potential confounders (comparison of crude and adjusted Odds-Ratios) and interaction factors (Wald's test of homogeneity).

These analyses will be performed using the Chi-squared test or Fisher's exact test for qualitative variables, and the Student's t test or the Mann-Whitney test (depending on the nature of the variable or the size of the sample) for quantitative variables. A significance level of less than 0.05 is considered associated.

Missing data and outliers

Missing data will be documented individually. In the case of multiple missing data or if there is evidence of bias in the missing data on the variables of interest, multiple imputation methods may be used at the study level to substitute missing values. Imputation will be performed if the proportion of missing data does not exceed 20%. If this is the case, the variable will be removed from the statistical analysis.

A sensitivity analysis will be performed to compare the raw database (including missing data) with the imputed data.

Outliers were be prevented by a blocking input mask, alert messages and an input guide. However, if new outliers are discovered after the quality checks and during the analyses, they will be corrected as missing data. The scripts allowing these corrections will be communicated.

For peer review only - http://bmjopen.bmj.com/site/about/guidelines.xhtml

For peer review only

Data management and archiving

Case report form

The case report form only included data necessary for participation in the TSARA project. All information required by the protocol is recorded in the anonymized case report forms. Data is collected as they are obtained in the laboratory and then at the patient's bedside, and recorded in these case report forms explicitly.

These case report forms are set up in each of the facilities using the electronic tablets equipped with Epicollect applications, including the TSARA project questionnaires. A instruction book to help the investigators use this tool was provided. The investigators are responsible for the accuracy, quality and relevance of all data entered, under the responsibility of the lead investigator.

These case report forms are associated with de-anonymized forms, in paper format, containing the first and last names of the patients enrolled, as well as an identification number shared between the paper and online forms. These second de-anonymized forms stay within the hospital and should allow possible corrections or deletions of data upon request of the patients enrolled.

Data management

Collected data are computerized by the epidemiologist in charge of the project. Electronic databases are anonymous and locked with a password known only by the scientific staff. These data will be kept for a minimum of 15 years after the end of the study.

Archiving

The sponsor will keep the study documents (protocol and annexes, possible amendments, information forms, case report form, statistical analysis plan and output and the final study report) for a minimum of 15 years. After this period, the sponsor will be consulted before any data are destroyed. Study-related documents and reports may be subject to audit or inspection by the sponsor and/or other authorized bodies. No relocation or destruction will be made without the consent of the sponsor. At the end of the 15 years, the sponsor will be consulted for destruction. All data, documents and reports will be subject to audit or inspection.

Confidentiality

For peer review only - http://bmjopen.bmj.com/site/about/guidelines.xhtml

All personal data concerning study participants will remain strictly confidential. To respect privacy, all participant details will be anonymous for the purpose of database preparation. Study subjects are coded with 4 numerical numbers.

Ethics and dissemination

Ethics

The TSARA study was approved by the National Ethics Committee in Madagascar on 24 January 2023 (n°07-MSANP/SG/AGMED/CNPV/CERBM).

Informed consent

Patients are fully and fairly informed, in understandable terms, of the objectives, their rights to refuse to participate in the study, or the opportunity to withdraw at any time.

All of this information is included on an information form given to the patient, and the patient's non-objection will be collected by the investigator before the start of inclusion and will be recorded in the patient's source file.

The information will be communicated orally in French or in Malagasy by the investigators. An information form is also available in paper format in French or in Malagasy. When conditions permit, if the eligible patient does not speak French or Malagasy, the investigators will seek to volunteer a third party to provide a translation (a relative of the patient, or available hospital personnel).

Regulatory compliance

The study will be conducted in accordance with applicable laws and regulations currently in place in Madagascar.

Withdrawal criteria

Subjects may request to withdraw from the study at any time, without explanation, for any reason. In the event of premature exit, the investigator should document the reasons as completely as possible.

For peer review only - http://bmjopen.bmj.com/site/about/guidelines.xhtml

Stopping the research study

The Mérieux Foundation reserves the right to discontinue the study at any time, if it is determined that the inclusion objectives are not met, or if the protocol is not respected in one or more participating institutions, or subject to other circumstances beyond the control of the Mérieux Foundation (e.g. political instability). In case of premature termination of the study in one of the institutions, the information will be transmitted by the sponsor to the Malagasy interlocutors.

Protocol amendments

In the eventuality of changes in the existing protocol that significantly affect the scope or the scientific quality of the investigation, an amendment containing a verbatim description of the changes and reference (date and number) to the submission that contained the original protocol will be submitted to the ethical committee for their approval.

Dissemination

Results and scientific reports that emerge from this study will be made publically available under the responsibility of the principal investigator in agreement with the associated investigators. Results will be reported following the guidelines from the STrengthening the Reporting of OBservational studies in Epidemiology consortium (www.strobe-statement.org) and STROBE-AMS (18). Publication rules will follow international recommendations. The findings will also be shared with national health and sport authorities.

Authorship will follow the guidelines established by the International Committee of Medical Journal Editors (https://www.icmje.org), which require substantive contributions to the design, conduct, and interpretation and reporting of an epidemiological study.

These data will help the Malagasy scientific societies of infectious diseases (SPIM), pediatrics (SMP) and microbiology (SOMABIO) to tailor antibiotic recommendations according to the resistance patterns as presented in the Figure 3.

For peer review only

For peer review only - http://bmjopen.bmj.com/site/about/guidelines.xhtml

Figure 3: Data flow between the different stakeholders

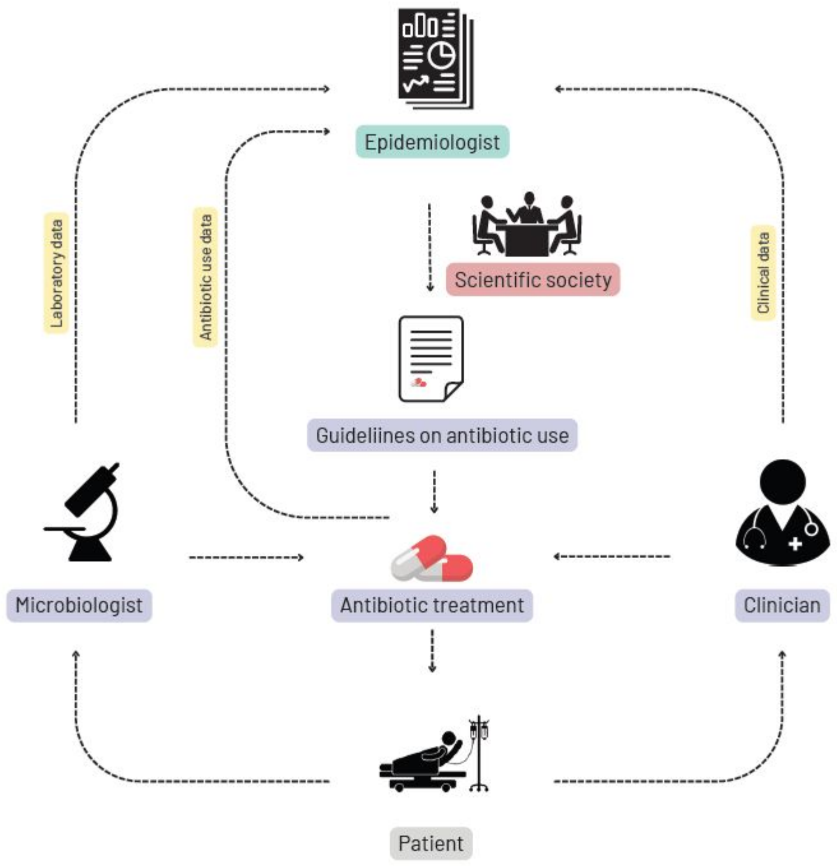

iew only

For peer review only - http://bmjopen.bmj.com/site/about/guidelines.xhtml

Discussion

The TSARA project will provide original results that could 1) allow a more precise epidemiological knowledge of bacterial infections, prescribed antibiotic therapies and local resistance levels ; 2) Inspire, correct and tailor local practices, including recommendations and guidelines for empiric antibiotic therapy according to local resistance patterns; 3) encourage communication and use of AST results with and between clinicians ; 4) allow monitoring of hospital antibiotic prescription (quantity, quality and spectrum, adequacy with bacteriological results) and 5) provide evidence to improve laboratory services and interactions with clinicians.

Surveillance is the primary strategy for tracking emerging drug resistance in the population, and thus provides a tool to pick up any unusual organisms early and perform appropriate action. Regular dissemination of AMR data are key to elaborate local, evidence-based recommendations and revise the AMR national policy.

<u>Shortcomings</u>

Implementing TSARA as an AMR laboratory and patient-based surveillance system presents some challenges. First, all the patients included in the study were hospitalized, and are self-paying for the majority of healthcare costs which induces a selection bias and may hamper the representativeness of the data. Mainly tertiary care hospitals belonging to the RESAMAD laboratory network were included due to their laboratory capacities, secondary and primary care centers were under-represented in TSARA. Consequently, results from the AMR data generated might be inflated as these facilities harbour more vulnerable and sick patients, who may be subject to higher antibiotic exposure. This tool, although innovative, is highly dependent on the capacity and the willingness of staff, on laboratory capacity, which can sometimes be heterogeneous within the hospitals and over the time, depending on the turnover of the staff and the availability of consumables in country. The TSARA project can be implemented only in a facility where laboratory services are functional and where bacterial cultures and AST are performed routinely. In addition, treatment could be influenced by a delay in obtaining the culture results. In Madagascar, as well as many LMICs, it is common to use diagnostic microbiology testing after treatment failure to improve on broad-spectrum antibiotics (19), which also overestimates local AMR rates. Negative perception of the laboratory capacity and associated costs could explain the unwillingness to perform microbiology testing by the clinicians.

For peer review only - http://bmjopen.bmj.com/site/about/guidelines.xhtml

No clear standardized definition of multidrug-resistant bacteria has been set up by Malagasy health authorities, which results in difficulties in the data interpretation and comparison. Data collection relies on paper-based data capture, where no electronic medical records currently exist in the facilities. The quality of data could be another obstacle to ensure validated results and might be exposed to intersite variability. Clinical data and follow up data could also be subject to a substantial proportion of missing data, as these are harder to retrieve than microbiological data. Lastly, there may be a low motivation of the investigator collecting the data as it could be perceived as added work with a limited incentive or reward for this work.

Opportunities

The collection of laboratory and clinical data remains a serious challenge in LMICs. In 2021, the Mérieux Foundation implemented the Lab Book 3.0 (20), a software which is a Laboratory Information System (LIS) and helps to computerize clinical laboratory data, in all the RESAMAD laboratories. This has improved data collection and ensures data quality.

Additionally, the use of information and technology through a digital tool could be the next step for TSARA. It would encourage a timely reporting of AMR and clinical data. Thus, it may incentivize scientific societies to adjust national antibiotic guidelines according to the local resistance patterns and therefore incite prescribers to better, more informed clinical decision-making and antibiotic prescription. Given the expansion of e-Health and connected devices, we could imagine this tool will be integrated in a prescription assistance device on a mobile application as a future solution to combat AMR. Presenting the clinician with the antibiotic therapy recommendation associated with the WHO AWaRe classification has already shown its deployment in the fight against AMR (6).

Moreover, since January 2022, any patient hospitalized with a life-threatening disease has all the hospital costs free of charge in Malagasy hospitals. Leadership and financial commitment from local health authorities has been invaluable (i.e. Direction of epidemiological surveillance and Response of the Ministry of Health), and is key to the sustainability of the project and its expansion across the country.

For peer review only

Conclusion

Thanks to TSARA, significant progress should be made in AMR surveillance and antimicrobial stewardship in Madagascar. TSARA is an innovative study, which captures prospectively microbiological as well as clinical data in Malagasy hospitals belonging to the RESAMAD network. This project will also participate in achieving the implementation of the national action plan on AMR in Madagascar. Assessing the impact and the efficiency of this project i.e. monitoring antibiotic prescriptions and checking their compliance with recommendations still need to be explored as clinicians' behaviors also drive AMR. Despite some challenges, future opportunities are encouraging for TSARA but rely on financial investment and strong leadership from health authorities to warrant the added value, the sustainability and the potential expansion of this project.

For peer review only

References

1. O'Neill J., Davies S., Rex J., White L.J., Murray R. Review on Antimicrobial Resistance, Tackling Drug-Resistant Infections Globally: Final Report and Recommendations. London, UK: Wellcome Trust and UK Government; 2016.
2. Murray CJ, Ikuta KS, Sharara F, Swetschinski L, Robles Aguilar G, Gray A, et al. Global burden of bacterial antimicrobial resistance in 2019: a systematic analysis. The Lancet. 2022 Jan;S0140673621027240.
3. WHO Global Action Plan for AMR. World Health Organization; 2015.
4. Pulcini C, Binda F, Lamkang AS, Trett A, Charani E, Goff DA, et al. Developing core elements and checklist items for global hospital antimicrobial stewardship programmes: a consensus approach. Clin Microbiol Infect. 2019 Jan;25(1):20–5.
5. Tebano G, Li G, Beovic B, Bielicki J, Brink A, Enani MA, et al. Essential and forgotten antibiotics: An inventory in low- and middle-income countries. Int J Antimicrob Agents. 2019 Sep;54(3):273–82.
6. Budd E, Cramp E, Sharland M, Hand K, Howard P, Wilson P, et al. Adaptation of the WHO Essential Medicines List for national antibiotic stewardship policy in England: being AWaRe. J Antimicrob Chemother. 2019 Nov 1;74(11):3384–9.
7. Mendelson M, Morris AM, Thursky K, Pulcini C. How to start an antimicrobial stewardship programme in a hospital. Clin Microbiol Infect [Internet]. 2019 Aug [cited 2023 Jul 3]; Available from: https://linkinghub.elsevier.com/retrieve/pii/S1198743X19304483
8. Elias C, Moja L, Mertz D, Loeb M, Forte G, Magrini N. Guideline recommendations and antimicrobial resistance: the need for a change. BMJ Open. 2017 Jul;7(7):e016264.
9. Cox JA, Vlieghe E, Mendelson M, Wertheim H, Ndegwa L, Villegas MV, et al. Antibiotic stewardship in low- and middle-income countries: the same but different? Clin Microbiol Infect. 2017 Nov;23(11):812–8.
10. World Bank Country Classification [Internet]. [cited 2023 Jul 3]. Available from: https://datahelpdesk.worldbank.org/knowledgebase/articles/906519-world-bank-country-and-lending-groups
11. RESAMAD [Internet]. [cited 2023 Jul 3]. Available from: https://www.fondation-merieux.org/projets/resamad/
12. Randriatsarafara FM, Ralamboson J, Rakotoarivelo R, Raherinandrasana A, Andrianasolo R. Consommation d'antibiotiques au Centre Hospitalier Universitaire d'Antananarivo : prévalence et défis stratégiques. Santé Publique. 2015;27(2):249.

For peer review only - http://bmjopen.bmj.com/site/about/guidelines.xhtml

13. Fondation Mérieux [Internet]. [cited 2023 Jul 3]. Available from: https://www.fondation-merieux.org/en/who-we-are/

14. Morency-Potvin P, Schwartz DN, Weinstein RA. Antimicrobial Stewardship: How the Microbiology Laboratory Can Right the Ship. Clin Microbiol Rev. 2017 Jan;30(1):381–407.

15. Protocol for Enhanced Isolate-Level Antimicrobial Resistance Surveillance in the Americas. Primary Phase: Bloodstream Infections [Internet]. Pan American Health Organization; 2021 [cited 2022 Feb 2]. Available from: https://iris.paho.org/handle/10665.2/53275

16. GRAM protocol [Internet]. [cited 2023 Jul 3] https://www.tropicalmedicine.ox.ac.uk/gram/research/global-burden-of-bacterial-antimicrobial-resistance/

17. And Schnall J, Rajkhowa A, Ikuta K, Rao P, Moore CE. Surveillance and monitoring of antimicrobial resistance: limitations and lessons from the GRAM project. BMC Med [Internet]. déc 2019 [cited 2023 Jul 3];17(1). Available from: https://bmcmedicine.biomedcentral.com/articles/10.1186/s12916-019-1412-8

18. Tacconelli E, Cataldo MA, Paul M, et al. STROBE-AMS: recommendations to optimise reporting of epidemiological studies on antimicrobial resistance and informing improvement in antimicrobial stewardship. BMJ Open 2016;6:e010134. doi:10.1136/bmjopen-2015- 010134

19. Krockow EM, Colman AM, Chattoe-Brown E, Jenkins DR, Perera N, Mehtar S, et al. Balancing the risks to individual and society: a systematic review and synthesis of qualitative research on antibiotic prescribing behaviour in hospitals. J Hosp Infect. 2019 Apr;101(4):428–39.

20. Lab Book [Internet]. [cited 2023 Jul 3]. Available from: https://www.lab-book.org/

For peer review only - http://bmjopen.bmj.com/site/about/guidelines.xhtml

Authors' contributions

M. Raad, FX. Babin, L. Raskine, M. Raberahona, S. Rasoanandrasana, M. Randria and C. Elias designed the study.

C. Elias and M. Raad drafted the manuscript.

All authors have revised and approved the final version of the manuscript.

Funding

The TSARA project has received funding from:

- Fondation Mérieux
- PFIZER ISID Research Grant

Competing interests

None declared.

Acknowledgements

The authors thank all the biologists and the local investigators of the RESAMAD network for their participation in the TSARA project.

The authors are very thankful for Luciana RAKOTOARISOA and Johannie RAKOTONIAINA for their support in the implementation of the project locally.

For peer review only - http://bmjopen.bmj.com/site/about/guidelines.xhtml

Supplementary material 1. TSARA microbiological questionnaire

Question	Name of the variable	Type of data	Format	Description
**Hospital where the sample was taken**	id_hosp	Single choice list	CHUJRB, CHUJRA, CHUMET, CHRR ANTSIRABE, CHU ANOSIALA, CHU MORAFENO , CHU PZAGA , CHU FIANARANTSOA, CHRR AMBOHIMIANDRA, CHU TULEAR	Hospital where the bacteriological sample was taken (hospital of inclusion)
**Patient identification number**	id_pat	Numeric	_ _ _ _ _ _ _ _ _ _	Patient's inclusion number
**Date of collection**	dt_pvt	Date	DD/MM/YYYY	Date of collection
**Date of receipt of the sample in the laboratory**	dt_rec_pvt	Date	DD/MM/YYYY	Date of receipt of the sample in the laboratory
**Date of bacteriological CR**	dt_cr_pvt	Date	DD/MM/YYYY	Date of delivery of the result to the patient or prescribing physician
**Type of sampling**	pvt_type	Single choice list	Blood culture, Intravascular device, Ascites fluid, Cerebrospinal fluid, Joint fluid, Respiratory specimen, Deep tissue specimen, Genital specimen, Urine, Neonatal specimen and placenta, Superficial pus, Deep tissue specimen, Stool culture (not screening), Other specimen, Unknown	Type of sampling performed
**Positive culture**	culture	Single choice list	Yes/No	Identification of a microorganism on the culture
**If yes, identified pathogen**	bact	Single choice list	List of bacteria	Identification of the micro-organism on the sample
**Nalidixic acid**	nalidixic_acid	Single choice list	S, I, R, NR	Sensitivity or resistance of the antibiotic tested
**Fusidic acid**	fusidic_acid	Single choice list	S, I, R, NR	Sensitivity or resistance of the antibiotic tested
**Amikacin**	amikacin	Single choice list	S, I, R, NR	Sensitivity or resistance of the antibiotic tested
**Amoxicillin**	amoxicillin	Single choice list	S, I, R, NR	Sensitivity or resistance of the antibiotic tested
**Amoxicillin-clavulanic acid**	amoxicillin_clav	Single choice list	S, I, R, NR	Sensitivity or resistance of the antibiotic tested
**Ampicillin**	ampicillin	Single choice list	S, I, R, NR	Sensitivity or resistance of the antibiotic tested
**Ampicillin-Sulbactam**	ampicillin_sulbactam	Single choice list	S, I, R, NR	Sensitivity or resistance of the antibiotic tested
**Azithromycin**	azithromycin	Single choice list	S, I, R, NR	Sensitivity or resistance of the antibiotic tested
**Aztreonam**	aztreonam	Single choice list	S, I, R, NR	Sensitivity or resistance of the antibiotic tested

For peer review only

**Cefadroxil**	cefadroxil	Single choice list	S, I, R, NR	Sensitivity or resistance of the antibiotic tested
**Cefalexin**	cefalexin	Single choice list	S, I, R, NR	Sensitivity or resistance of the antibiotic tested
**Cefalotin**	cefalotin	Single choice list	S, I, R, NR	Sensitivity or resistance of the antibiotic tested
**Cefamandole**	cefamandole	Single choice list	S, I, R, NR	Sensitivity or resistance of the antibiotic tested
**Cefazolin**	cefazolin	Single choice list	S, I, R, NR	Sensitivity or resistance of the antibiotic tested
**Cefepime**	cefpime	Single choice list	S, I, R, NR	Sensitivity or resistance of the antibiotic tested
**Cefixime**	cefixime	Single choice list	S, I, R, NR	Sensitivity or resistance of the antibiotic tested
**Cefotaxime**	cefotaxime	Single choice list	S, I, R, NR	Sensitivity or resistance of the antibiotic tested
**Cefoxitin**	cefoxitin	Single choice list	S, I, R, NR	Sensitivity or resistance of the antibiotic tested
**Cefpodoxime**	cefpodoxime	Single choice list	S, I, R, NR	Sensitivity or resistance of the antibiotic tested
**Ceftazidime**	ceftazidime	Single choice list	S, I, R, NR	Sensitivity or resistance of the antibiotic tested
**Ceftriaxone**	ceftriaxone	Single choice list	S, I, R, NR	Sensitivity or resistance of the antibiotic tested
**Cefuroxime**	cefuroxime	Single choice list	S, I, R, NR	Sensitivity or resistance of the antibiotic tested
**Chloramphenicol**	chloramphenicol	Single choice list	S, I, R, NR	Sensitivity or resistance of the antibiotic tested
**Chlortetracycline**	chlortetracycline	Single choice list	S, I, R, NR	Sensitivity or resistance of the antibiotic tested
**Ciprofloxacin**	ciprofloxacin	Single choice list	S, I, R, NR	Sensitivity or resistance of the antibiotic tested
**Clarithromycin**	clarithromycin	Single choice list	S, I, R, NR	Sensitivity or resistance of the antibiotic tested
**Clindamycin**	clindamycin	Single choice list	S, I, R, NR	Sensitivity or resistance of the antibiotic tested
**Cloxacillin**	cloxacillin	Single choice list	S, I, R, NR	Sensitivity or resistance of the antibiotic tested
**Colistin**	colistin	Single choice list	S, I, R, NR	Sensitivity or resistance of the antibiotic tested
**Daptomycin**	daptomycin	Single choice list	S, I, R, NR	Sensitivity or resistance of the antibiotic tested
**Doxycycline**	doxycycline	Single choice list	S, I, R, NR	Sensitivity or resistance of the antibiotic tested
**Ertapeneme**	ertapeneme	Single choice list	S, I, R, NR	Sensitivity or resistance of the antibiotic tested
**Erythromycin**	erythromycin	Single choice list	S, I, R, NR	Sensitivity or resistance of the antibiotic tested
**Fosfomycin**	fosfomycin	Single choice list	S, I, R, NR	Sensitivity or resistance of the antibiotic tested
**Gentamicin**	gentamicin	Single choice list	S, I, R, NR	Sensitivity or resistance of the antibiotic tested
**Imipeneme**	imipeneme	Single choice list	S, I, R, NR	Sensitivity or resistance of the antibiotic tested
**Josamycin**	josamycin	Single choice list	S, I, R, NR	Sensitivity or resistance of the antibiotic tested
**Levofloxacin**	levofloxacin	Single choice list	S, I, R, NR	Sensitivity or resistance of the antibiotic tested

For peer review only

**Lincomycin**	lincomycin	Single choice list	S, I, R, NR	Sensitivity or resistance of the antibiotic tested
**Linezolid**	linezolide	Single choice list	S, I, R, NR	Sensitivity or resistance of the antibiotic tested
**Meropeneme**	meropeneme	Single choice list	S, I, R, NR	Sensitivity or resistance of the antibiotic tested
**Metronidazole**	metronidazole	Single choice list	S, I, R, NR	Sensitivity or resistance of the antibiotic tested
**Midecamycin**	midecamycin	Single choice list	S, I, R, NR	Sensitivity or resistance of the antibiotic tested
**Minocycline**	minocycline	Single choice list	S, I, R, NR	Sensitivity or resistance of the antibiotic tested
**Moxifloxacin**	moxifloxacin	Single choice list	S, I, R, NR	Sensitivity or resistance of the antibiotic tested
**Neomycin**	neomycin	Single choice list	S, I, R, NR	Sensitivity or resistance of the antibiotic tested
**Netilmycin**	netilmycin	Single choice list	S, I, R, NR	Sensitivity or resistance of the antibiotic tested
**Nifuroxazide**	nifuroxazide	Single choice list	S, I, R, NR	Sensitivity or resistance of the antibiotic tested
**Nitrofurantoin**	nitrofurantoin	Single choice list	S, I, R, NR	Sensitivity or resistance of the antibiotic tested
**Norfloxacin**	norfloxacin	Single choice list	S, I, R, NR	Sensitivity or resistance of the antibiotic tested
**Ofloxacin**	ofloxacin	Single choice list	S, I, R, NR	Sensitivity or resistance of the antibiotic tested
**Oxacillin**	oxacillin	Single choice list	S, I, R, NR	Sensitivity or resistance of the antibiotic tested
**Pefloxacin**	pefloxacin	Single choice list	S, I, R, NR	Sensitivity or resistance of the antibiotic tested
**Piperacillin**	piperacillin	Single choice list	S, I, R, NR	Sensitivity or resistance of the antibiotic tested
**Piperacillin-Tazobactam**	piperacillin_tazo	Single choice list	S, I, R, NR	Sensitivity or resistance of the antibiotic tested
**Pivmecillinam**	pivmecillinam	Single choice list	S, I, R, NR	Sensitivity or resistance of the antibiotic tested
**Polymyxin**	polymyxin	Single choice list	S, I, R, NR	Sensitivity or resistance of the antibiotic tested
**Pristinamycin**	pristinamycin	Single choice list	S, I, R, NR	Sensitivity or resistance of the antibiotic tested
**Rifampicin**	rifampin	Single choice list	S, I, R, NR	Sensitivity or resistance of the antibiotic tested
**Spectinomycin**	spectinomycin	Single choice list	S, I, R, NR	Sensitivity or resistance of the antibiotic tested
**Streptomycin**	streptomycin	Single choice list	S, I, R, NR	Sensitivity or resistance of the antibiotic tested
**Sulfamethoxazole**	sulfamethoxazole	Single choice list	S, I, R, NR	Sensitivity or resistance of the antibiotic tested
**Tedizolide**	tedizolide	Single choice list	S, I, R, NR	Sensitivity or resistance of the antibiotic tested
**Teicoplanin**	teicoplanin	Single choice list	S, I, R, NR	Sensitivity or resistance of the antibiotic tested
**Telithromycin**	telithromycin	Single choice list	S, I, R, NR	Sensitivity or resistance of the antibiotic tested
**Temocillin**	temocillin	Single choice list	S, I, R, NR	Sensitivity or resistance of the antibiotic tested
**Ticarcillin**	ticarcillin	Single choice list	S, I, R, NR	Sensitivity or resistance of the antibiotic tested

For peer review only

**Ticarcillin-clavulanic acid**	ticarcillin_clav	Single choice list	S, I, R, NR	Sensitivity or resistance of the antibiotic tested
**Tigecycline**	tigecycline	Single choice list	S, I, R, NR	Sensitivity or resistance of the antibiotic tested
**Tobramycin**	tobramycin	Single choice list	S, I, R, NR	Sensitivity or resistance of the antibiotic tested
**Vancomycin**	vancomycin	Single choice list	S, I, R, NR	Sensitivity or resistance of the antibiotic tested

For peer review only

Supplementary material 2. TSARA Clinical Questionnaire

Question	Name of the variable	Type of data	Format	Description
**Date of visit in the room**	dt_visit	Date	DD/MM/YYYY	Date of patient/clinician visit to clinical service
**Gender**	gender	Unique choice	Male, Female	Sex of the patient
**Date of birth**	dt_naiss	Date	DD/MM/YYYY	Patient's date of birth
**Age**	age	Numeric	_ _ _	Age of patient at hospital admission in years
**Size**	size	Numeric	_ _ _	Patient height (cm) at admission
**Weight**	weight	Numeric	_ _ _	Patient weight (kg) at admission
**Status at birth**	status_naiss	Single choice list	Premature, Born at term	Premature status at the birth of the child
**Patient's residence**	place_resid	Single choice list	Urban, Rural	Patient's primary place of residence on admission
**Patient's region of residence**	reg_resid	Text	_ _ _ _ _ _ _ _ _ _ _ _ _ _ _	Patient's primary area of residence on admission
**Date of admission to hospital**	dt_adm_hosp	Date	DD/MM/YYYY	Date of hospital entry (TSARA participant)
**Patient's origin**	from	Single choice list	Home, Transfer to anotherhospital, Unknown	Patient's origin before admission to hospital
**Diagnosis at admission**	diag_adm	Single choice list	Infectious, Non-infectious, Unknown	Patient diagnosis on admission to hospital
**Type of service**	type_service	Single choice list	Medicine, Surgery, Intensive Care, Gyneco-Obstetrics,Pediatrics, Other	Type of service where the sample was taken
**Respiratory rate (beats per minute)**	freq_resp	Numeric	_ _ _	Patient's respiratory rate on admission
**Heart rate (beats per minute)**	freq_card	Numeric	_ _ _	Patient's heart rate on admission
**Systolic blood pressure (mmHg)**	pa_sys	Numeric	_ _ _	Systolic blood pressure on admission
**Disorder of consciousness**	awareness	Single choice list	Yes/No	Presence of disturbed consciousness at admission
**Peripheral venous access**	vvp	Single choice list	Yes/No	Placement of a PVV between admission and collection
**Central venous access**	vvc	Single choice list	Yes/No	Placement of a CVC between admission and

For peer review only

				collection
**Urinary catheterization**	probe_uro	Single choice list	Yes/No	Placement of a urinary catheter between admission andsampling
**Oro-tracheal intubation**	intubation	Single choice list	Yes/No	Intubation performed between admission and collection
**Reason for collection**	motif_pvt	Multiple choice list	List of reason for samples	Reason for collection
**Date of onset of symptoms**	dt_sympt	Date	DD/MM/YYYY	Date of onset of the first symptoms for which the sample was taken
**Antibiotic history within 3 months prior to admission**	atcd_atb	Single choice list	Yes/No	Use of ATB within 3 months of hospital admission
**If yes, specify ATBs**	type_atcd_atb	Multiple choice list	List of antibiotics	If yes, specify the type of ATB
**Antibiotic therapy during the stay before sampling**	atb_hosp	Single choice list	Yes/No	Antibiotic treatment prescribed between admission to hospital and sampling
**If yes, specify ATBs**	type_atb_hosp	Multiple choice list	List of antibiotics	If yes, specify the type of ATB
**Antibiotic therapy after receiving the antibiogram**	atb_post_atbg	Single choice list	Yes/No	Antibiotic treatment prescribed after receiving the antibiogram
**If yes, specify ATBs**	type_atb_post_atbg	Multiple choice list	List of antibiotics	If yes, specify the type of ATB
**History of hospitalization in the 6 months preceding thehospitalization**	atcd_hosp	Single choice list	Yes/No	History of hospitalization in the 6 months preceding thehospitalization
**History of surgery in the 6 months preceding hospitalization**	atcd_chir	Single choice list	Yes/No	History of surgery in the 6 months preceding hospitalization
**Comorbidities**	comorbidities	Multiple choice list	List of comorbidities	Presence of clinical comorbidities at admission
**Other comorbidities**	other_comorbidity	Free text		Other comorbidities

For peer review only

Supplementary material 3. TSARA III follow-up questionnaire

Question	Name of the variable	Type of data	Format	Description
**Date of discharge from hospital**	dt_end_hosp	Date	DD/MM/YYYY	End date of hospital stay
**Exit diagnosis**	diag_hosp	Single choice list	List of diagnosis	
**Patient status at discharge**	hosp_output	Single choice list	RAD, Deceased, Transfer to another hospital	Patient status at the end of the hospital stay

For peer review only

Implementation of an antibiotic resistance surveillance tool in Madagascar, the TSARA project: a prospective, observational, multicenter, hospital-based study protocol.

Journal:	*BMJ Open*
Manuscript ID	bmjopen-2023-078504.R1
Article Type:	Protocol
Date Submitted by the Author:	12-Jan-2024
Complete List of Authors:	Elias, Christelle; Hospices Civils de Lyon, Service Hygiène, Epidémiologie et Prévention; Université Claude Bernard Lyon 1, Public Health, Epidemiology & Evolutionary Ecology of Infectious Diseases, Centre International de Recherche<br>Raad, Mathieu; Fondation Mérieux, Direction des Opérations Internationales<br>Rasoanandrasana, Saida; Hôpital Befelatanana, Service de Biologie<br>Raherinandrasana, Antso Hasina; Faculté de Médecine de l'Université d'Antananarivo, Département de Santé Publique<br>Andriananja, Volatiana; Hôpital Befelatanana, Service des Maladies Infectieuses<br>Raberahona, Mihaja; University Hospital Joseph Raseta Befelatanana, Infectious Diseases<br>Moore, Catrin; Saint George's Hospital, Centre for Neonatal and Paediatric Infection<br>Randria, Mamy; Hôpital Befelatanana, Service de Biologie<br>Raskine, Laurent; Fondation Mérieux, Direction des Opérations Internationales<br>Vanhems, Philippe; Hospices Civils de Lyon, Service d'Hygiène, Epidémiologie et Prévention, Lyon, France; Université Claude Bernard Lyon 1, 2. Public Health, Epidemiology & Evolutionary Ecology of Infectious Diseases (PHE3ID) team, Centre International de Recherche en Infectiologie (CIRI), Inserm U1111, CNRS UMR5308<br>Babin, François-Xavier; Fondation Mérieux, Direction des Opérations Internationales
**Primary Subject Heading**:	Infectious diseases
Secondary Subject Heading:	Epidemiology, Diagnostics
Keywords:	BACTERIOLOGY, Protocols & guidelines < HEALTH SERVICES ADMINISTRATION & MANAGEMENT, Diagnostic microbiology < INFECTIOUS DISEASES, Epidemiology < INFECTIOUS DISEASES

For peer review only - http://bmjopen.bmj.com/site/about/guidelines.xhtml

Implementation of an antibiotic resistance surveillance tool in Madagascar, the TSARA project: a prospective, observational, multicenter, hospital-based study protocol.

C.Elias[1,2], M. Raad[3], S. Rasoanandrasana[4], AH. Raherinandrasana[5,], V. Andriananja[6], M. Raberahona[6], C.E. Moore[7], M. Randria[4], L. Raskine[3], P Vanhems[1,2], FX. Babin[3]

1. Service Hygiène et Epidémiologie, Hospices Civils de Lyon, Lyon, France
2. Public Health, Epidemiology & Evolutionary Ecology of Infectious Diseases (PHE3ID) team, Centre International de Recherche en Infectiologie (CIRI), Inserm U1111, CNRS UMR5308, ENS de Lyon, Université Claude Bernard Lyon 1, Lyon, France
3. Direction des Opérations Internationales, Fondation Mérieux, Lyon, France
4. Service de Biologie, Hôpital Befelatanana, Antananarivo, Madagascar
5. Département de Santé Publique, Faculté de Médecine de l'Université d'Antananarivo, Madagascar
6. Service des Maladies Infectieuses, Hôpital Befelatanana, Antananarivo, Madagascar
7. Centre for Neonatal and Paediatric Infection, St. George's, University of London, London

Corresponding Author:

Dr Christelle ELIAS

Service Hygiène et Epidémiologie, Hospices Civils de Lyon, Lyon

165 Chemin du Grand Revoyet, 69645 PIERRE-BENITE Cedex

christelle.elias@chu-lyon.fr

Phone: +33 4 78 86 12 73

Keywords: Antimicrobial Resistance, AMR, Antimicrobial Stewardship, Developing countries, Guidelines, Hospital, Low- and- middle-income countries, Madagascar, Surveillance

For peer review only

For peer review only - http://bmjopen.bmj.com/site/about/guidelines.xhtml

Abstract

Introduction: Antimicrobial resistance (AMR) has become a significant public health threat. Without any interventions, it has been modelled that AMR will account for an estimated 10 million deaths annually by 2050, this mainly affects low- and- middle-income countries. AMR has a systemic negative perspective affecting the overall healthcare system down to the patient's personal outcome. In response to this issue, the WHO urged countries to provide antimicrobial stewardship programs (ASP). ASP in hospitals are a vital component of national action plans for AMR, and have been shown to significantly reduce AMR, in particular in low-income countries such as Madagascar.

As part of an ASP, AMR surveillance provides essential information needed to guide medical practice. We developed an AMR surveillance tool - TSARA - with the support of the Mérieux Foundation. TSARA combines bacteriological and clinical information to provide a better understanding of the scope and the effects of AMR in Madagascar, where no such surveillance tool exists.

Methods and analysis: A prospective, observational, hospital-based study was carried out for data collection using a standardized data collection tool, called TSARA deployed in 2023 in 10 hospitals in Madagascar participating to the national Malagasy laboratory network (RESAMAD). Any hospitalized patient where the clinician decided to take a bacterial sample is included. As a prospective study, individual isolate-level data and antimicrobial susceptibility information on pathogens were collected routinely from the bacteriology laboratory and compiled with clinical information retrieved from face-to-face interviews with the patient and completed using medical records where necessary. Analysis of the local ecology, resistance rates and antibiotic prescription patterns were collected.

Ethics and dissemination: This protocol obtained ethical approval from the Malagasy Ethical Committee n°07-MSANP/SG/AGMED/CNPV/CERBM on January 24, 2023. Findings generated were shared to national health stakeholders, microbiologists, members of the RESAMAD Network and the Malagasy academic society of Infectious diseases.

For peer review only - http://bmjopen.bmj.com/site/about/guidelines.xhtml

Strengths and limitations of this study

- TSARA is a prospective, observational and multicentre protocol capturing data from hospitalized patients.
- TSARA is an AMR surveillance tool that compiles microbiological and clinical data, including information on antibiotic prescriptions implemented in Malagasy hospitals belonging to the RESAMAD network.
- The data collection is performed on an electronic tablet which limits data entry bias.
- The TSARA project is highly dependent on the infrastructure and the available resources in Malagasy laboratories.

For peer review only

For peer review only - http://bmjopen.bmj.com/site/about/guidelines.xhtml

Introduction

Antimicrobial resistance (AMR) is a global health threat associated with a poor prognosis and an increased mortality in the population infected with resistant bacteria. In the coming decades, deaths attributable to AMR will overcome the mortality due to non-communicable diseases through the lack of treatment options (1). Recent data from the Global Burden of Disease (2) suggested that the impact of AMR on health is even more important than previous estimations (1). To address the increasing problem, the World Health Organization (WHO) published the Global Action Plan on AMR in 2015 (3) and urged countries to build national action plans aiming to prioritise interventions to combat AMR rapidly. One of the objectives was to implement and strengthen surveillance systems to monitor AMR as a first step of an antimicrobial stewardship program (ASP) (4), in particular in hospital settings where high levels of resistance occur. In addition to these measures, the WHO also advocates for the implementation of antibiotic management policies adapted to local resistance patterns in hospitals (5–8). Setting up an ASP is challenging, especially in low- and- middle-income countries (LMICs) due to the lack of infrastructure, good quality data and governance (7,9). For a country like Madagascar, where robust healthcare facilities, infrastructures and laboratories are very scarce, collecting resistance data remains a challenge.

Madagascar is the fifth largest island in the world, but is considered a low-income country according to the World Bank (10). Health coverage in Madagascar is extremely limited and the patient has to directly pay for the majority of health care (i.e. diagnostics, treatment, consumables, surgery). Most drugs - especially antibiotics - are dispensed only with a medical prescription. However, in Madagascar, it is very common to deliver such medicines “Over The Counter” and antibiotics can be sold easily outside hospital settings, for those who can afford them, without any medical prescription. Lack or substandard infection control practices contribute significantly to the spread of bacterial resistance, both in the community (through AMR/antibiotics in wastewater) and in hospitals (hospital hygiene and nosocomial transmission). The culture of systematic treatment against an infectious agent encourages the misuse and the overconsumption of antibiotics, prescribed or not. At the hospital level, AMR has a greater effect, as patients are in hospital for several days, receive more care, have invasive procedures performed and thus are more at risk of transmission of bacteria and healthcare-associated infections. Appropriate hygiene measures and standard precautions are difficult to implement in low-resources settings, where there is for instance an average of five hospital beds per room in a Malagasy university hospital.

For peer review only - http://bmjopen.bmj.com/site/about/guidelines.xhtml

Outside of the RESAMAD laboratories, there are very few laboratories than can perform microbiology cultures and antimicrobial susceptibility testing (AST) in Madagascar and the burdened cost remains prohibitive for many patients. Targeted antibiotic therapy is therefore rarely prescribed and antibiotic treatment often remains empirical without any de-escalation. In this context of low access to microbiology laboratory combined with high rates of bacterial resistance at the national level, practitioners use broad-spectrum therapies, increasing the selection pressure on the bacteria and creating emerging resistances. Knowledge of the local resistance patterns is essential to adapt the clinical decision-making and have locally adapted recommendations (8). Table 1 provides a SWOT (Strengths, Weaknesses, Opportunities and Threats) analysis as an overview of what is currently in place and the starting points of the implementation of an AMR surveillance tool in Madagascar.

Table 1. SWOT analysis of the implementation of an AMR surveillance tool in hospitals in Madagascar

**Strengths**	**Weaknesses**
– Laboratory network RESAMAD (11) currently in place, already transferring data to the WHO through the GLASS-AMR module<br>– Active support of international stakeholders<br>– Identification of highly motivated Malagasy practitioners willing to work on AMR and ASP<br>– Existence of several scientific societies (infectious diseases, pediatrics, medical biology[1])	– Little knowledge on the local epidemiology of AMR<br>– Collection of antibiograms not performed on a daily basis<br>– Lack of visibility on antibiotic prescriptions in hospitals and their efficiency<br>– Implementation only doable where good laboratories exist<br>– Bacteriology results sometimes delivered to the prescriber in a paper format
**Opportunities**	**Threats**
– Capture high level antibiotic use in hospitals<br>– Existing antibiotic recommendations in place, but they are not adapted to local resistance patterns (12)<br>– Existing AMR protocols including the surveillance of antimicrobial use	– Challenging implementation<br>– Substantial missing data<br>– Personnel require training<br>– Lack of funding<br>– Political instability<br>– Climate disorders

[1] Société de Pathologie Infectieuse de Madagascar, Société Malgache de Pédiatrie, SOMABIO

For peer review only - http://bmjopen.bmj.com/site/about/guidelines.xhtml

In Madagascar as well as in multiple developing countries, the Mérieux Foundation has been strengthening clinical laboratories in the fight against infectious diseases for decades to enhance the quality and access to clinical laboratories aiming at preventing infections and improving patient care (13). The Mérieux Foundation has been investing on the fight against AMR supporting countries to develop infrastructures, laboratories, personnel training and building a laboratory network to strengthen laboratory-based surveillance of infectious diseases. As a result, in 2022, 14 Malagasy laboratories were in the RESAMAD (Réseau des Laboratoires à Madagascar) laboratory network, they were all able to perform bacteriology diagnosis, this was created in 2007 and has risen to 27 laboratories across the country to date (RESAMAD - Fondation Mérieux (fondation-merieux.org)).

Given this context, and the challenge to combat AMR in healthcare settings, ASP in hospitals are a vital component of national action plans for AMR, and have been shown to significantly reduce AMR, in particular in low-income country such as Madagascar (9,14). As part of an ASP, AMR surveillance provides essential information needed to guide medical practice. An AMR surveillance tool called TSARA (Technique de Surveillance Actualisée de la Résistance aux Antimicrobiens) was developed thanks to the support of the Mérieux Foundation, with the objective of combining patient data with laboratory and epidemiological surveillance data to provide a better understanding of the scope and the effects of AMR in Madagascar, where no such surveillance tool was available, in order to treat patients more effectively. It will allow monitoring and follow-up compared to the scarce existing prescribing data (15). The TSARA project also aligns with the antimicrobial surveillance program led by the Direction of epidemiological surveillance and Response of the Ministry of Health in the context of One Health and in collaboration with other Ministries to tackle AMR in Madagascar. TSARA also answers one of the objective of the Malagasy national action plan on AMR (16).

For peer review only - http://bmjopen.bmj.com/site/about/guidelines.xhtml

Objectives

One of the most important benefits of AMR surveillance at the pathogen level is to achieve a standardized, comparable, and easily validated data collection method for quality data that is used to implement better patient treatment and hospital/national guidelines (17). The data allows an optimization of the patient care by adapting treatment guidelines according the patient demographics and type of infection, taking into consideration of the circulating pathogens and local resistance patterns. The primary objective is:

1. To institute a systematic approach towards microbiological data and clinical data collection and analysis of antimicrobial susceptibility information to optimize antimicrobial therapy in Malagasy hospitals.

The secondary objectives are:

1. To establish timely reporting of information on pathogens of interest and their antimicrobial susceptibility profiles to medical doctors;
2. To allow monitoring of the hospital antibiotic prescription (quantity, quality and spectrum, concordance with bacteriological results);
3. To provide data to the laboratory quality assessment and identify areas for improvement.

For peer review only

For peer review only - http://bmjopen.bmj.com/site/about/guidelines.xhtml

Methods and analysis

Study design and setting

Study design

TSARA is a prospective, observational, multicenter, hospital-based study based on a standardized data collection tool deployed in ten facilities with both adult and pediatric patients in Madagascar (Figure 1), collecting microbiological, demographic, prescription and clinical data in one place.

Figure 1. Map of the laboratories and implementation sites of TSARA in Madagascar

Outcome measures

The outcomes of interest will be categorized into behavioural, clinical and microbiological outcomes. Behavioural outcomes include changes in prescribing practices among prescribers and compliance to AMS protocols. In the TSARA project, we consider an adapted antibiotic prescription as a behavioral outcome, defined as:

1. A prescription of an antibiotic therapy consistent with the national antibiotic guidelines, or
2. A prescription of an antibiotic therapy considering the laboratory results including antibiotic susceptibility testing results.

Then, a metric will be used to describe the number and the proportion of the adapted antibiotic prescriptions.

Clinical outcomes include mortality defined as death occurring during the patient's hospital stay or the length of stay defined as the duration between the admission date and discharge date from the hospital.
Microbiological outcomes include a description of the bacteria isolated by culture, bacterial resistance rates as well as the delay between the sample being collected and the laboratory results communicated to the prescriber (in days).

Setting and laboratory capacity

For peer review only

For peer review only - http://bmjopen.bmj.com/site/about/guidelines.xhtml

All laboratories participating in the TSARA project are part of the nationwide RESAMAD network in Madagascar (11). Description of the infrastructure in each hospital included in the TSARA project is presented in Supplmentary material 1.

These laboratories perform diagnostics and antimicrobial susceptibility testing for infectious diseases, and have the capacity to perform phenotypic confirmation of the presence of resistance. To participate in TSARA, laboratories have to meet minimal quality standards requirements defined by the RESAMAD network and participate in an external quality assessment program. The laboratories also have the required infrastructure, equipment, supplies and resources to perform AST following EUCAST standards. The reporting of results to clinicians are made by qualified and trained technicians supervised by clinical microbiologists. The Mérieux Foundation performs regular training to laboratory staff and support the purchase of consumables (i.e. Petri dish, antibiotic disks) and reagents.

<u>Study population</u>

The population of the study includes all patients (adults and children) arriving at the hospital who are inpatients and have bacterial samples taken by the physician and sent to the laboratory.

Inclusion criteria

All patients arriving at each hospital, who have a bacterial culture performed are included in the study. All positive and negative bacteriological samples from hospitalized patients in one of the participating institutions from January 2023 onwards will be included.

Exclusion criteria

The following criteria will be excluded from the TSARA project:

- All non-bacteriological specimens (virological, fungal, parasitic)
- All samples from patients not hospitalized >24 hours (emergency, consultation, outpatient hospitalization)
- All environmental samples (water, air, surfaces, medical devices)
- All post-mortem samples
- All samples reported as contaminated defined as results that do not contribute to clinical decision-making

For peer review only - http://bmjopen.bmj.com/site/about/guidelines.xhtml

- All specimens for which culture was not possible
- All specimens with laboratory non-compliance
- All the patients or legal guardians who object to be included in the TSARA project
- All patients with severe cognitive impairment
- All patients who do not have a full understanding of the French or Malagasy language

Participant timeline

Enrolled patients will be followed up during their hospital stay until discharge or death. Information on the vital status (alive, deceased, discharged or transferred to another hospital) will be collected.

Patient and public involvement

Patients and the public were not involved in the design of this study.

Data collection

Individual isolate-level data and AST on pathogens were collected routinely from the bacteriology laboratory using a first questionnaire (Supplementary material 2). These data were compiled using a second questionnaire with basic clinical, demographic, and epidemiological information (including current antibiotic prescription) retrieved daily through face-to-face interviews with the patient and completed by medical records where necessary (Supplementary material 3). A third questionnaire was then filled in regarding follow-up data (Supplementary material 4). The data retrieved from TSARA meet the criteria and are linked to the GRAM project that seeks to estimate the burden of AMR worldwide (17, 18).

To enhance data collection and limit data entry bias, data were directly captured on a tablet connected to the Epicollect5® platform (https://five.epicollect.net). Data were collected by Malagasy investigators (medical interns, biologists or clinical research associates) using tablets dedicated to this project, each of whom received specific training on the TSARA methodology prior to the start of the study. The investigator will examine the bacteriological samples daily, or as frequently as possible. A new eligible sample corresponds to a new inclusion in the TSARA project.

For peer review only - http://bmjopen.bmj.com/site/about/guidelines.xhtml
For peer review only

Statistical methods

Sample size

TSARA is a surveillance project, as such there is no calculation on the number of subjects needed *a priori*. Statistical analyses will be adapted *a posteriori* according to the total number of inclusions during the project period and the power needed to obtain valid results.

Description of the statistical methods

Any anomalies in the data will be checked with medical records during data cleaning. Data will be validated by quality control to identify outliers and inconsistencies by means of consistency tests (e.g. filling in the inclusion criteria, date of admission to hospital later than the date of admission to the department, date of death later than the date of onset of symptoms).

Descriptive summary tables with numbers and frequencies will be drawn up. Similarly, visual representations of the variables may be made to detect irregular or missing values. Any changes to the data will be documented and recorded separately from the raw database. Recoded variables will also be filled in (e.g. age categories).

A flow chart will be developed to describe the number of eligible, included and excluded patients and samples in our study. A descriptive analysis of the entire baseline population will be undertaken to identify the baseline characteristics of the study population (the specimen, bacterial, clinical and patient levels).

Categorical variables will be reported as frequencies and percentages of the total population. Quantitative variables will be described using the mean and standard deviation if the distribution is normal, or the median and interquartile range if the distribution does not follow the normal distribution. The range with the minimum and maximum values will be filled in. For each variable, the proportion of missing values will be indicated.

Stratified analyses will also be provided to present results by facility, department, type of sample, and bacteria of interest (including species, and antibiotic resistance of interest) as well as by type of antibiotics prescribed. In addition, given the fact that data are collected for the adult and pediatric population, a stratified analysis will be performed for both populations.

A two-group comparison using univariate and then multivariate analysis and a logistic regression will be performed to estimate the associations between an adapted antibiotic therapy and the other variables. The Odds-Ratio and p-value of each test will be reported. Only the variables with a p-value

For peer review only - http://bmjopen.bmj.com/site/about/guidelines.xhtml

lower than 0.2 will be retained to perform the multivariate analyses. In addition to the presentation of the multivariate analyses, the most efficient multivariate model will be presented after a top-down selection of only those variables with a p-value less than 0.05. Stratified analyses will be conducted to identify potential confounders (comparison of crude and adjusted Odds-Ratios) and interaction factors (Wald's test of homogeneity).

These analyses will be performed using the Chi-squared test or Fisher's exact test for qualitative variables, and the Student's t test or the Mann-Whitney test (depending on the nature of the variable or the size of the sample) for quantitative variables. A significance level of less than 0.05 is considered associated.

Missing data and outliers

Missing data will be documented individually. In the case of multiple missing data or if there is evidence of bias in the missing data on the variables of interest, multiple imputation methods may be used at the study level to substitute missing values. Imputation will be performed if the proportion of missing data does not exceed 20%. If this is the case, the variable will be removed from the statistical analysis.

A sensitivity analysis will be performed to compare the raw database (including missing data) with the imputed data.

Outliers were be prevented by a blocking input mask, alert messages and an input guide. However, if new outliers are discovered after the quality checks and during the analyses, they will be corrected as missing data. The scripts allowing these corrections will be communicated.

<u>Data management and archiving</u>

Case report form

The case report form only includes data necessary for participation in the TSARA project. All information required by the protocol is recorded in the anonymized case report forms. Data are collected as they are obtained in the laboratory and then at the patient's bedside, and recorded in these case report forms explicitly.

These case report forms are set up in each of the facilities using the electronic tablets equipped with Epicollect applications, including the TSARA project questionnaires. An instruction book to help the

For peer review only - http://bmjopen.bmj.com/site/about/guidelines.xhtml

For peer review only

investigators use this tool was provided. The investigators are responsible for the accuracy, quality and relevance of all data entered, under the responsibility of the lead investigator.

These case report forms are associated with de-anonymized forms, in paper format, containing the first and last names of the patients enrolled, as well as an identification number shared between the paper and online forms. These second de-anonymized forms stay within the hospital and should allow possible corrections or deletions of data upon request of the patients enrolled.

Data management

Collected data are computerized by the epidemiologist in charge of the project. Electronic databases are anonymous and locked with a password known only by the scientific staff. These data will be kept for a minimum of 15 years after the end of the study.

Archiving

The sponsor will keep the study documents (protocol and annexes, possible amendments, information forms, case report form, statistical analysis plan and output and the final study report) for a minimum of 15 years. After this period, the sponsor will be consulted before any data are destroyed. Study-related documents and reports may be subject to audit or inspection by the sponsor and/or other authorized bodies. No relocation or destruction will be made without the consent of the sponsor. At the end of the 15 years, the sponsor will be consulted for destruction. All data, documents and reports will be subject to audit or inspection.

Confidentiality

All personal data on the study participants will remain strictly confidential. To respect their privacy and for confidentiality, all participant details will be anonymous for the purpose of database preparation. Study subjects are coded with 4 numerical numbers.

Ethics and dissemination

Ethics

For peer review only - http://bmjopen.bmj.com/site/about/guidelines.xhtml

For peer review only

The TSARA study was approved by the National Ethics Committee in Madagascar on 24 January 2023 (n°07-MSANP/SG/AGMED/CNPV/CERBM).

Informed consent

Patients are fully and fairly informed, in understandable terms, of the objectives, their rights to refuse to participate in the study, or the opportunity to withdraw at any time.

All of this information is included on an information form given to the patient, and the patient's agreement will be collected by the investigator being included and will be recorded in the patient's source file.

The information will be communicated orally in French or in Malagasy by the investigators. An information form is also available in paper format in French and in Malagasy. When conditions permit, if the eligible patient does not speak French or Malagasy, the investigators will seek to volunteer a third party to provide a translation (a relative of the patient, or available hospital personnel).

Regulatory compliance

The study will be conducted in accordance with applicable laws and regulations currently in place in Madagascar.

Withdrawal criteria

Subjects may request to withdraw from the study at any time, without explanation, for any reason. In the event of premature exit, the investigator should document the reasons as completely as possible.

Stopping the research study

The Mérieux Foundation reserves the right to discontinue the study at any time, if it is determined that the inclusion objectives are not met, or if the protocol is not respected in one or more participating institutions, or subject to other circumstances beyond the control of the Mérieux Foundation (e.g. political instability). In case of premature termination of the study in one of the institutions, the information will be transmitted by the sponsor to the Malagasy interlocutors.

For peer review only

For peer review only - http://bmjopen.bmj.com/site/about/guidelines.xhtml

Protocol amendments

In the eventuality of changes in the existing protocol that significantly affect the scope or the scientific quality of the investigation, an amendment containing a verbatim description of the changes and reference (date and number) to the submission that contained the original protocol will be submitted to the ethical committee for their approval.

Dissemination

Results and scientific reports that emerge from this study will be made publically available. This is under the responsibility of the principal investigator in agreement with the associated investigators. Results will be reported following the guidelines from the STrengthening the Reporting of OBservational studies in Epidemiology consortium (www.strobe-statement.org) and STROBE-AMS (19). Publication rules will follow international recommendations. The findings will also be shared with national health and sport authorities.

Authorship will follow the guidelines established by the International Committee of Medical Journal Editors (https://www.icmje.org), which require substantive contributions to the design, conduct, and interpretation and reporting of an epidemiological study.

These data will help the Malagasy scientific societies of infectious diseases (SPIM), pediatrics (SMP) and microbiology (SOMABIO) to tailor antibiotic recommendations according to the resistance patterns as presented in the Figure 2.

Figure 2: Data flow between the different stakeholders

For peer review only - http://bmjopen.bmj.com/site/about/guidelines.xhtml

Discussion

The TSARA project will provide original results that could 1) allow a more precise epidemiological knowledge of bacterial infections, prescribed antibiotic therapies and local resistance levels ; 2) Inspire, correct and tailor local practices, including recommendations and guidelines for empiric antibiotic therapy according to local resistance patterns ; 3) encourage communication and use of AST results with and between clinicians ; 4) allow monitoring of hospital antibiotic prescription (quantity, quality and spectrum, concordance with bacteriological results) and 5) provide evidence to improve laboratory services and interactions with clinicians.

Surveillance is the primary strategy for tracking emerging drug resistance in the population, and thus provides a tool to pick up any unusual organisms early and perform appropriate action. Regular dissemination of AMR data is key to elaborate local, evidence-based recommendations and revise the AMR national policy.

Shortcomings

Implementing TSARA as an AMR laboratory and patient-based surveillance system presents some challenges. First, all the patients included in the study were hospitalized, and are self-paying for the majority of healthcare costs which induces a selection bias and may hamper the representativeness of the data. Mainly tertiary care hospitals belonging to the RESAMAD laboratory network were included due to their laboratory capacities, secondary and primary care centers were under-represented in TSARA. Consequently, results from the AMR data generated might be inflated as these facilities harbour more vulnerable and sick patients, who may have previously taken antibiotics and been in hospital and therefore be subject to higher antibiotic exposure. Additionally, clinicians may prescribe antibiotics in the absence of microbiological documentation or for the treatment of non-bacterial infections, even though such practices could contribute to elevated rates of antibiotic resistance. This tool, although innovative, is highly dependent on the capacity and the willingness of staff, on laboratory capacity, which can sometimes be heterogeneous within the hospitals and over the time, depending on the turnover of the staff and the availability of consumables in country. The TSARA project can be implemented only in a facility where laboratory services are functional, robust and where bacterial cultures and AST are performed routinely. In addition, patient treatment could be influenced by a delay in obtaining the culture results. In Madagascar, as well as many LMICs, it is common to use diagnostic microbiology testing after treatment failure to improve on broad-spectrum antibiotics (20), which also overestimates local AMR rates. Negative perception of the laboratory

For peer review only - http://bmjopen.bmj.com/site/about/guidelines.xhtml

capacity and associated costs could explain the unwillingness to perform microbiology testing by the clinicians.

No clear standard definition of multidrug-resistant bacteria has been set up by Malagasy health authorities, which results in difficulties in the data interpretation and comparison. Data collection currently relies on paper-based data capture, where no electronic medical records currently exist in the facilities. The quality of data may be another obstacle to getting validated results and there might be exposed to intersite variability. Clinical data and follow up data could also be subject to a substantial proportion of missing data, as these are harder to retrieve than microbiological data. Lastly, the investigator may have a lack of motivation to collect the data as it could be perceived as added work with a limited incentive or reward.

Opportunities

The culture to collect of laboratory and clinical data is a serious challenge in LMICs. In 2021, the Mérieux Foundation implemented the Lab Book 3.0 (21), a Laboratory Information System (LIS) which helps to computerize clinical laboratory data, in all the RESAMAD laboratories. This has both improved data collection and ensures data quality.

Additionally, the use of information and technology through a digital tool could be the next step for TSARA. It would encourage a timely reporting of AMR and clinical data ensuring the correct patient treatment. Thus, it may incentivize scientific societies to adjust national antibiotic guidelines according to the local resistance patterns and hence support prescribers to make better, more informed clinical decisions and antibiotic prescription. Given the expansion of e-Health and connected devices, we imagine this tool to be integrated in a prescription assistance device on a mobile application as a future solution to combat AMR. Presenting the clinician with the antibiotic therapy recommendation associated with the WHO AWaRe classification has already shown its deployment in the fight against AMR (6).

Furthermore, starting from January 2022, a change in the patient payment has occurred where any patient admitted to a Malagasy hospital with a life-threatening disease has received all the initial hospital care at no cost. Leadership and financial commitment from local health authorities has been invaluable (i.e. Direction of epidemiological surveillance and Response of the Ministry of Health), and is key to the sustainability of the project and its expansion across the country.

Thanks to TSARA, significant progress should be made in AMR surveillance and antimicrobial stewardship in Madagascar. This project will also participate in supporting the implementation of the

For peer review only - http://bmjopen.bmj.com/site/about/guidelines.xhtml

national action plan on AMR in Madagascar. Assessing the impact and the efficiency of this project i.e. monitoring antibiotic prescriptions and checking their compliance with recommendations still need to be explored as clinicians' behaviors also drive AMR. Despite some challenges, future opportunities are encouraging for TSARA but rely on financial investment and strong leadership from health authorities to warrant the added value, the sustainability and the potential expansion of this project.

For peer review only

For peer review only - http://bmjopen.bmj.com/site/about/guidelines.xhtml

References

1. O'Neill J., Davies S., Rex J., White L.J., Murray R. Review on Antimicrobial Resistance, Tackling Drug-Resistant Infections Globally: Final Report and Recommendations. London, UK: Wellcome Trust and UK Government; 2016.
2. Murray CJ, Ikuta KS, Sharara F, Swetschinski L, Robles Aguilar G, Gray A, et al. Global burden of bacterial antimicrobial resistance in 2019: a systematic analysis. The Lancet. 2022 Jan;S0140673621027240.
3. WHO Global Action Plan for AMR. World Health Organization; 2015.
4. Pulcini C, Binda F, Lamkang AS, Trett A, Charani E, Goff DA, et al. Developing core elements and checklist items for global hospital antimicrobial stewardship programmes: a consensus approach. Clin Microbiol Infect. 2019 Jan;25(1):20–5.
5. Tebano G, Li G, Beovic B, Bielicki J, Brink A, Enani MA, et al. Essential and forgotten antibiotics: An inventory in low- and middle-income countries. Int J Antimicrob Agents. 2019 Sep;54(3):273–82.
6. Budd E, Cramp E, Sharland M, Hand K, Howard P, Wilson P, et al. Adaptation of the WHO Essential Medicines List for national antibiotic stewardship policy in England: being AWaRe. J Antimicrob Chemother. 2019 Nov 1;74(11):3384–9.
7. Mendelson M, Morris AM, Thursky K, Pulcini C. How to start an antimicrobial stewardship programme in a hospital. Clin Microbiol Infect [Internet]. 2019 Aug [cited 2023 Jul 3]; Available from: https://linkinghub.elsevier.com/retrieve/pii/S1198743X19304483
8. Elias C, Moja L, Mertz D, Loeb M, Forte G, Magrini N. Guideline recommendations and antimicrobial resistance: the need for a change. BMJ Open. 2017 Jul;7(7):e016264.
9. Cox JA, Vlieghe E, Mendelson M, Wertheim H, Ndegwa L, Villegas MV, et al. Antibiotic stewardship in low- and middle-income countries: the same but different? Clin Microbiol Infect. 2017 Nov;23(11):812–8.
10. World Bank Country Classification [Internet]. [cited 2023 Jul 3]. Available from: https://datahelpdesk.worldbank.org/knowledgebase/articles/906519-world-bank-country-and-lending-groups
11. RESAMAD [Internet]. [cited 2023 Jul 3]. Available from: https://www.fondation-merieux.org/projets/resamad/
12. Randriatsarafara FM, Ralamboson J, Rakotoarivelo R, Raherinandrasana A, Andrianasolo R. Consommation d'antibiotiques au Centre Hospitalier Universitaire d'Antananarivo : prévalence et défis stratégiques. Santé Publique. 2015;27(2):249.

For peer review only - http://bmjopen.bmj.com/site/about/guidelines.xhtml

13. Fondation Mérieux [Internet]. [cited 2023 Jul 3]. Available from: https://www.fondation-merieux.org/en/who-we-are/
14. Morency-Potvin P, Schwartz DN, Weinstein RA. Antimicrobial Stewardship: How the Microbiology Laboratory Can Right the Ship. Clin Microbiol Rev. 2017 Jan;30(1):381–407.
15. Protocol for Enhanced Isolate-Level Antimicrobial Resistance Surveillance in the Americas. Primary Phase: Bloodstream Infections [Internet]. Pan American Health Organization; 2021 [cited 2022 Feb 2]. Available from: https://iris.paho.org/handle/10665.2/53275
16. Plan d'action national pour combattre la résistance aux antimicrobiens 2019-2023 [cited 2023 Dec 22] Available from: https://cdn.who.int/media/docs/default-source/antimicrobial-resistance/amr-spc-npm/nap-library/madagascar_2019_2023.pdf?sfvrsn=cf290d1c_1&download=true
17. GRAM protocol [Internet]. [cited 2023 Jul 3] https://www.tropicalmedicine.ox.ac.uk/gram/research/global-burden-of-bacterial-antimicrobial-resistance/
18. And Schnall J, Rajkhowa A, Ikuta K, Rao P, Moore CE. Surveillance and monitoring of antimicrobial resistance: limitations and lessons from the GRAM project. BMC Med [Internet]. déc 2019 [cited 2023 Jul 3];17(1). Available from: https://bmcmedicine.biomedcentral.com/articles/10.1186/s12916-019-1412-8
19. Tacconelli E, Cataldo MA, Paul M, et al. STROBE-AMS: recommendations to optimise reporting of epidemiological studies on antimicrobial resistance and informing improvement in antimicrobial stewardship. BMJ Open 2016;6:e010134. doi:10.1136/bmjopen-2015- 010134
20. Krockow EM, Colman AM, Chattoe-Brown E, Jenkins DR, Perera N, Mehtar S, et al. Balancing the risks to individual and society: a systematic review and synthesis of qualitative research on antibiotic prescribing behaviour in hospitals. J Hosp Infect. 2019 Apr;101(4):428–39.
21. Lab Book [Internet]. [cited 2023 Jul 3]. Available from: https://www.lab-book.org/

For peer review only

For peer review only - http://bmjopen.bmj.com/site/about/guidelines.xhtml

Authors' contributions

C. Elias, M. Raad, FX. Babin, L. Raskine, M. Raberahona, S. Rasoanandrasana, C. E. Moore and M. Randria designed the study.

C. Elias and M. Raad drafted the manuscript.

C.Elias, S. Rasoanandrasana, AH. Raherinandrasana, V. Andriananja, and L Raskine participated in the acquisition of data.

C.Elias, M. Raad, S. Rasoanandrasana, AH. Raherinandrasana, V. Andriananja, M. Raberahona, C.E. Moore M. Randria, L. Raskine, P Vanhems and FX. Babin have revised and approved the final version of the manuscript.

Funding

The TSARA project has received funding from:

- Fondation Mérieux
- PFIZER ISID Research Grant n°63019455

Competing interests

None declared.

Acknowledgements

The authors thank all the biologists and the local investigators of the RESAMAD network for their participation in the TSARA project.

The authors are very thankful for Luciana RAKOTOARISOA and Johannie RAKOTONIAINA for their support in the implementation of the project locally.

For peer review only - http://bmjopen.bmj.com/site/about/guidelines.xhtml

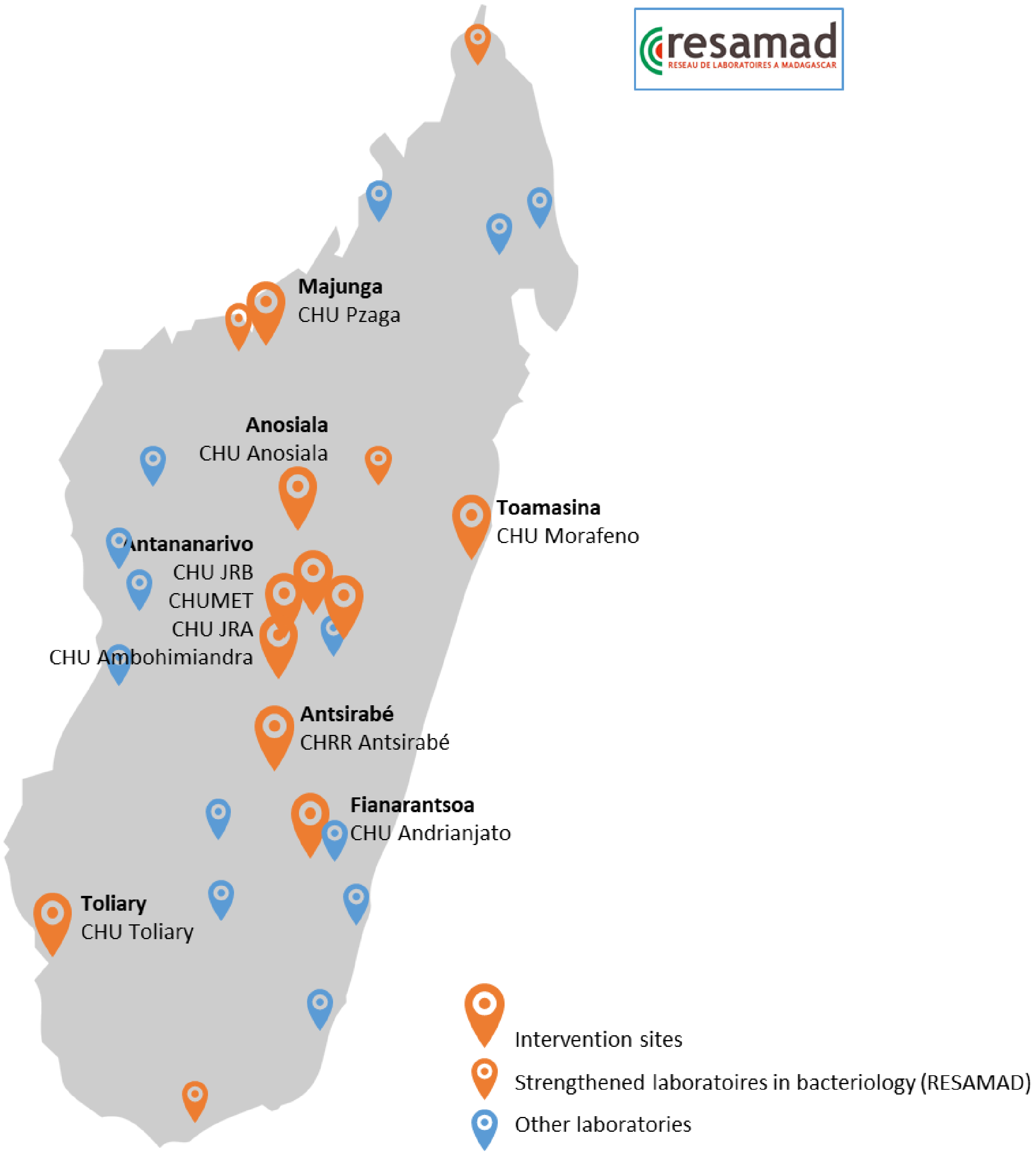
resamad
RESEAU DE LABORATOIRES A MADAGASCAR
Majunga
CHU Pzaga
Anosiala
CHU Anosiala
Toamasina
CHU Morafeno
Antananarivo
CHU JRB
CHUMET
CHU JRA
CHU Ambohimiandra
Antsirabé
CHRR Antsirabé
Fianarantsoa
CHU Andrianjato
Toliary
CHU Toliary
Intervention sites
Strengthened laboratoires in bacteriology (RESAMAD)
Other laboratories

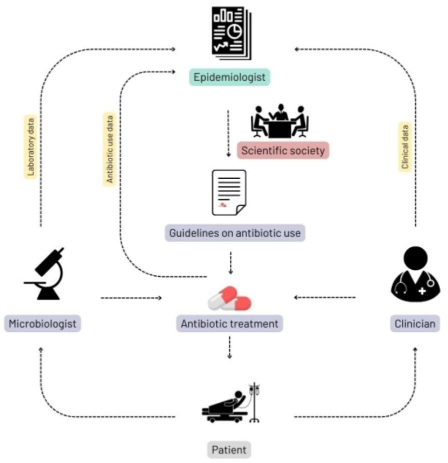

Figure 2. Data flow between the different stakeholders

162x91mm (300 x 300 DPI)

Supplementary material 1. Description of facilities included in the TSARA project

	CHU[a] Joseph Ravoahangy Andrianavalona (JRA)	CHU Joseph Raseta Befalatanana (JRB)	CHU Mère-Enfant Tsaralalana (MET)	CHU Ambohimiandra	CHU Anosiala	CHRR[b] Antsirabe	CHU Andrianjato	CHU Pzaga	CHU Morafeno	CHU Toliary
**City**	Antananarivo	Antananarivo	Antananarivo	Antananarivo	Anosiala	Antsirabe	Fianarantsoa	Mahajanga	Toamasina	Toliary
**Type of hospital[c]**	Tertiary	Tertiary	Tertiary	Tertiary	Tertiary	Secondary	Tertiary	Tertiary	Tertiary	Tertiary
**University/Regional hospital**	Teaching	Teaching	Teaching	Teaching	Teaching	Regional	Teaching	Teaching	Teaching	Teaching
**Hospital size (number of beds)**	595 beds	450 beds	200 beds	44 beds	180 beds	70 beds	120 beds	303 beds	120 beds	40 beds
**Hospital ownership**	Public	Public	Public	Public	Public	Public	Public	Public	Public	Public
**Type of population**	Adult	Adult	Pediatrics	Adult & Pediatrics	Adult	Adult	Adult	Adult	Adult	Adult
**Presence of ICU[d]**	Yes	Yes	Yes	Yes	Yes	Yes	Yes	Yes	Yes	Yes
**Strengthened bacteriology laboratory**	Yes	Yes	Yes	Yes	Yes	Yes	Yes	Yes	Yes	Yes
**Diagnostic AST[e] method**	Disk diffusion (EUCAST v. 2021)	Disk diffusion (EUCAST v. 2021)	Disk diffusion (EUCAST v. 2021)	Disk diffusion (EUCAST v. 2021)	Disk diffusion (EUCAST v. 2021)	Disk diffusion (EUCAST v. 2021)	Disk diffusion (EUCAST v. 2021)	Disk diffusion (EUCAST v. 2021)	Disk diffusion (EUCAST v. 2021)	Disk diffusion (EUCAST v. 2021)
**Local guidelines on the use of antibiotics in place**	None	None	None	None	None	None	None	None	None	None

[a]CHU : Centre hospitalier Universitaire (Teaching hospital)
[b]CHRR : Centre Hospitalier de Référence régionale (Regional hospital)
[c]https://www.who.int/publications/i/item/WHO-EMP-IAU-2018.01
[d]ICU: Intensive Care Unit
[e]AST: Antibiotic susceptibility testing

For peer review only

Supplementary material 2. TSARA microbiological questionnaire

Question	Name of the variable	Type of data	Format	Description
**Hospital where the sample was taken**	id_hosp	Single choice list	CHUJRB, CHUJRA, CHUMET, CHRR ANTSIRABE, CHU ANOSIALA, CHU MORAFENO , CHU PZAGA , CHU FIANARANTSOA, CHRR AMBOHIMIANDRA, CHU TULEAR	Hospital where the bacteriological sample was taken (hospital of inclusion)
**Patient identification number**	id_pat	Numeric	_ _ _ _ _ _ _ _ _ _	Patient's inclusion number
**Date of collection**	dt_pvt	Date	DD/MM/YYYY	Date of collection
**Date of receipt of the sample in the laboratory**	dt_rec_pvt	Date	DD/MM/YYYY	Date of receipt of the sample in the laboratory
**Date of bacteriological CR**	dt_cr_pvt	Date	DD/MM/YYYY	Date of delivery of the result to the patient or prescribing physician
**Type of sampling**	pvt_type	Single choice list	Blood culture, Intravascular device, Ascites fluid, Cerebrospinal fluid, Joint fluid, Respiratory specimen, Deep tissue specimen, Genital specimen, Urine, Neonatal specimen and placenta, Superficial pus, Deep tissue specimen, Stool culture (not screening), Other specimen, Unknown	Type of sampling performed
**Positive culture**	culture	Single choice list	Yes/No	Identification of a microorganism on the culture
**If yes, identified pathogen**	bact	Single choice list	List of bacteria	Identification of the micro-organism on the sample
**Nalidixic acid**	nalidixic_acid	Single choice list	S, I, R, NR	Sensitivity or resistance of the antibiotic tested
**Fusidic acid**	fusidic_acid	Single choice list	S, I, R, NR	Sensitivity or resistance of the antibiotic tested
**Amikacin**	amikacin	Single choice list	S, I, R, NR	Sensitivity or resistance of the antibiotic tested
**Amoxicillin**	amoxicillin	Single choice list	S, I, R, NR	Sensitivity or resistance of the antibiotic tested
**Amoxicillin-clavulanic acid**	amoxicillin_clav	Single choice list	S, I, R, NR	Sensitivity or resistance of the antibiotic tested
**Ampicillin**	ampicillin	Single choice list	S, I, R, NR	Sensitivity or resistance of the antibiotic tested
**Ampicillin-Sulbactam**	ampicillin_sulbactam	Single choice list	S, I, R, NR	Sensitivity or resistance of the antibiotic tested
**Azithromycin**	azithromycin	Single choice list	S, I, R, NR	Sensitivity or resistance of the antibiotic tested
**Aztreonam**	aztreonam	Single choice list	S, I, R, NR	Sensitivity or resistance of the antibiotic tested

For peer review only

**Cefadroxil**	cefadroxil	Single choice list	S, I, R, NR	Sensitivity or resistance of the antibiotic tested
**Cefalexin**	cefalexin	Single choice list	S, I, R, NR	Sensitivity or resistance of the antibiotic tested
**Cefalotin**	cefalotin	Single choice list	S, I, R, NR	Sensitivity or resistance of the antibiotic tested
**Cefamandole**	cefamandole	Single choice list	S, I, R, NR	Sensitivity or resistance of the antibiotic tested
**Cefazolin**	cefazolin	Single choice list	S, I, R, NR	Sensitivity or resistance of the antibiotic tested
**Cefepime**	cefpime	Single choice list	S, I, R, NR	Sensitivity or resistance of the antibiotic tested
**Cefixime**	cefixime	Single choice list	S, I, R, NR	Sensitivity or resistance of the antibiotic tested
**Cefotaxime**	cefotaxime	Single choice list	S, I, R, NR	Sensitivity or resistance of the antibiotic tested
**Cefoxitin**	cefoxitin	Single choice list	S, I, R, NR	Sensitivity or resistance of the antibiotic tested
**Cefpodoxime**	cefpodoxime	Single choice list	S, I, R, NR	Sensitivity or resistance of the antibiotic tested
**Ceftazidime**	ceftazidime	Single choice list	S, I, R, NR	Sensitivity or resistance of the antibiotic tested
**Ceftriaxone**	ceftriaxone	Single choice list	S, I, R, NR	Sensitivity or resistance of the antibiotic tested
**Cefuroxime**	cefuroxime	Single choice list	S, I, R, NR	Sensitivity or resistance of the antibiotic tested
**Chloramphenicol**	chloramphenicol	Single choice list	S, I, R, NR	Sensitivity or resistance of the antibiotic tested
**Chlortetracycline**	chlortetracycline	Single choice list	S, I, R, NR	Sensitivity or resistance of the antibiotic tested
**Ciprofloxacin**	ciprofloxacin	Single choice list	S, I, R, NR	Sensitivity or resistance of the antibiotic tested
**Clarithromycin**	clarithromycin	Single choice list	S, I, R, NR	Sensitivity or resistance of the antibiotic tested
**Clindamycin**	clindamycin	Single choice list	S, I, R, NR	Sensitivity or resistance of the antibiotic tested
**Cloxacillin**	cloxacillin	Single choice list	S, I, R, NR	Sensitivity or resistance of the antibiotic tested
**Colistin**	colistin	Single choice list	S, I, R, NR	Sensitivity or resistance of the antibiotic tested
**Daptomycin**	daptomycin	Single choice list	S, I, R, NR	Sensitivity or resistance of the antibiotic tested
**Doxycycline**	doxycycline	Single choice list	S, I, R, NR	Sensitivity or resistance of the antibiotic tested
**Ertapeneme**	ertapeneme	Single choice list	S, I, R, NR	Sensitivity or resistance of the antibiotic tested
**Erythromycin**	erythromycin	Single choice list	S, I, R, NR	Sensitivity or resistance of the antibiotic tested
**Fosfomycin**	fosfomycin	Single choice list	S, I, R, NR	Sensitivity or resistance of the antibiotic tested
**Gentamicin**	gentamicin	Single choice list	S, I, R, NR	Sensitivity or resistance of the antibiotic tested
**Imipeneme**	imipeneme	Single choice list	S, I, R, NR	Sensitivity or resistance of the antibiotic tested
**Josamycin**	josamycin	Single choice list	S, I, R, NR	Sensitivity or resistance of the antibiotic tested
**Levofloxacin**	levofloxacin	Single choice list	S, I, R, NR	Sensitivity or resistance of the antibiotic tested

For peer review only

**Lincomycin**	lincomycin	Single choice list	S, I, R, NR	Sensitivity or resistance of the antibiotic tested
**Linezolid**	linezolide	Single choice list	S, I, R, NR	Sensitivity or resistance of the antibiotic tested
**Meropeneme**	meropeneme	Single choice list	S, I, R, NR	Sensitivity or resistance of the antibiotic tested
**Metronidazole**	metronidazole	Single choice list	S, I, R, NR	Sensitivity or resistance of the antibiotic tested
**Midecamycin**	midecamycin	Single choice list	S, I, R, NR	Sensitivity or resistance of the antibiotic tested
**Minocycline**	minocycline	Single choice list	S, I, R, NR	Sensitivity or resistance of the antibiotic tested
**Moxifloxacin**	moxifloxacin	Single choice list	S, I, R, NR	Sensitivity or resistance of the antibiotic tested
**Neomycin**	neomycin	Single choice list	S, I, R, NR	Sensitivity or resistance of the antibiotic tested
**Netilmycin**	netilmycin	Single choice list	S, I, R, NR	Sensitivity or resistance of the antibiotic tested
**Nifuroxazide**	nifuroxazide	Single choice list	S, I, R, NR	Sensitivity or resistance of the antibiotic tested
**Nitrofurantoin**	nitrofurantoin	Single choice list	S, I, R, NR	Sensitivity or resistance of the antibiotic tested
**Norfloxacin**	norfloxacin	Single choice list	S, I, R, NR	Sensitivity or resistance of the antibiotic tested
**Ofloxacin**	ofloxacin	Single choice list	S, I, R, NR	Sensitivity or resistance of the antibiotic tested
**Oxacillin**	oxacillin	Single choice list	S, I, R, NR	Sensitivity or resistance of the antibiotic tested
**Pefloxacin**	pefloxacin	Single choice list	S, I, R, NR	Sensitivity or resistance of the antibiotic tested
**Piperacillin**	piperacillin	Single choice list	S, I, R, NR	Sensitivity or resistance of the antibiotic tested
**Piperacillin-Tazobactam**	piperacillin_tazo	Single choice list	S, I, R, NR	Sensitivity or resistance of the antibiotic tested
**Pivmecillinam**	pivmecillinam	Single choice list	S, I, R, NR	Sensitivity or resistance of the antibiotic tested
**Polymyxin**	polymyxin	Single choice list	S, I, R, NR	Sensitivity or resistance of the antibiotic tested
**Pristinamycin**	pristinamycin	Single choice list	S, I, R, NR	Sensitivity or resistance of the antibiotic tested
**Rifampicin**	rifampin	Single choice list	S, I, R, NR	Sensitivity or resistance of the antibiotic tested
**Spectinomycin**	spectinomycin	Single choice list	S, I, R, NR	Sensitivity or resistance of the antibiotic tested
**Streptomycin**	streptomycin	Single choice list	S, I, R, NR	Sensitivity or resistance of the antibiotic tested
**Sulfamethoxazole**	sulfamethoxazole	Single choice list	S, I, R, NR	Sensitivity or resistance of the antibiotic tested
**Tedizolide**	tedizolide	Single choice list	S, I, R, NR	Sensitivity or resistance of the antibiotic tested
**Teicoplanin**	teicoplanin	Single choice list	S, I, R, NR	Sensitivity or resistance of the antibiotic tested
**Telithromycin**	telithromycin	Single choice list	S, I, R, NR	Sensitivity or resistance of the antibiotic tested
**Temocillin**	temocillin	Single choice list	S, I, R, NR	Sensitivity or resistance of the antibiotic tested
**Ticarcillin**	ticarcillin	Single choice list	S, I, R, NR	Sensitivity or resistance of the antibiotic tested

For peer review only

**Ticarcillin-clavulanic acid**	ticarcillin_clav	Single choice list	S, I, R, NR	Sensitivity or resistance of the antibiotic tested
**Tigecycline**	tigecycline	Single choice list	S, I, R, NR	Sensitivity or resistance of the antibiotic tested
**Tobramycin**	tobramycin	Single choice list	S, I, R, NR	Sensitivity or resistance of the antibiotic tested
**Vancomycin**	vancomycin	Single choice list	S, I, R, NR	Sensitivity or resistance of the antibiotic tested

For peer review only

Supplementary material 3. TSARA Clinical Questionnaire

Question	Name of the variable	Type of data	Format	Description
**Date of visit in the room**	dt_visit	Date	DD/MM/YYYY	Date of patient/clinician visit to clinical service
**Gender**	gender	Unique choice	Male, Female	Sex of the patient
**Date of birth**	dt_naiss	Date	DD/MM/YYYY	Patient's date of birth
**Age**	age	Numeric	_ _ _	Age of patient at hospital admission in years
**Size**	size	Numeric	_ _ _	Patient height (cm) at admission
**Weight**	weight	Numeric	_ _ _	Patient weight (kg) at admission
**Status at birth**	status_naiss	Single choice list	Premature, Born at term	Premature status at the birth of the child
**Patient's residence**	place_resid	Single choice list	Urban, Rural	Patient's primary place of residence on admission
**Patient's region of residence**	reg_resid	Text	_ _ _ _ _ _ _ _ _ _ _ _ _ _ _	Patient's primary area of residence on admission
**Date of admission to hospital**	dt_adm_hosp	Date	DD/MM/YYYY	Date of hospital entry (TSARA participant)
**Patient's origin**	from	Single choice list	Home, Transfer to anotherhospital, Unknown	Patient's origin before admission to hospital
**Diagnosis at admission**	diag_adm	Single choice list	Infectious, Non-infectious, Unknown	Patient diagnosis on admission to hospital
**Type of service**	type_service	Single choice list	Medicine, Surgery, Intensive Care, Gyneco-Obstetrics,Pediatrics, Other	Type of service where the sample was taken
**Respiratory rate (beats per minute)**	freq_resp	Numeric	_ _ _	Patient's respiratory rate on admission
**Heart rate (beats per minute)**	freq_card	Numeric	_ _ _	Patient's heart rate on admission
**Systolic blood pressure (mmHg)**	pa_sys	Numeric	_ _ _	Systolic blood pressure on admission
**Disorder of consciousness**	awareness	Single choice list	Yes/No	Presence of disturbed consciousness at admission
**Peripheral venous access**	vvp	Single choice list	Yes/No	Placement of a PVV between admission and collection
**Central venous access**	vvc	Single choice list	Yes/No	Placement of a CVC between admission and

For peer review only

				collection
**Urinary catheterization**	probe_uro	Single choice list	Yes/No	Placement of a urinary catheter between admission andsampling
**Oro-tracheal intubation**	intubation	Single choice list	Yes/No	Intubation performed between admission and collection
**Reason for collection**	motif_pvt	Multiple choice list	List of reason for samples	Reason for collection
**Date of onset of symptoms**	dt_sympt	Date	DD/MM/YYYY	Date of onset of the first symptoms for which the sample was taken
**Antibiotic history within 3 months prior to admission**	atcd_atb	Single choice list	Yes/No	Use of ATB within 3 months of hospital admission
**If yes, specify ATBs**	type_atcd_atb	Multiple choice list	List of antibiotics	If yes, specify the type of ATB
**Antibiotic therapy during the stay before sampling**	atb_hosp	Single choice list	Yes/No	Antibiotic treatment prescribed between admission to hospital and sampling
**If yes, specify ATBs**	type_atb_hosp	Multiple choice list	List of antibiotics	If yes, specify the type of ATB
**Antibiotic therapy after receiving the antibiogram**	atb_post_atbg	Single choice list	Yes/No	Antibiotic treatment prescribed after receiving the antibiogram
**If yes, specify ATBs**	type_atb_post_atbg	Multiple choice list	List of antibiotics	If yes, specify the type of ATB
**History of hospitalization in the 6 months preceding thehospitalization**	atcd_hosp	Single choice list	Yes/No	History of hospitalization in the 6 months preceding thehospitalization
**History of surgery in the 6 months preceding hospitalization**	atcd_chir	Single choice list	Yes/No	History of surgery in the 6 months preceding hospitalization
**Comorbidities**	comorbidities	Multiple choice list	List of comorbidities	Presence of clinical comorbidities at admission
**Other comorbidities**	other_comorbidity	Free text		Other comorbidities

For peer review only

Supplementary material 4. TSARA III follow-up questionnaire

Question	Name of the variable	Type of data	Format	Description
**Date of discharge from hospital**	dt_end_hosp	Date	DD/MM/YYYY	End date of hospital stay
**Exit diagnosis**	diag_hosp	Single choice list	List of diagnosis	
**Patient status at discharge**	hosp_output	Single choice list	RAD, Deceased, Transfer to another hospital	Patient status at the end of the hospital stay

For peer review only

BMJ Open

Implementation of an antibiotic resistance surveillance tool in Madagascar, the TSARA project: a prospective, observational, multicenter, hospital-based study protocol.

Journal:	*BMJ Open*
Manuscript ID	bmjopen-2023-078504.R2
Article Type:	Protocol
Date Submitted by the Author:	29-Feb-2024
Complete List of Authors:	Elias, Christelle; Hospices Civils de Lyon, Service Hygiène, Epidémiologie et Prévention; Université Claude Bernard Lyon 1, Public Health, Epidemiology & Evolutionary Ecology of Infectious Diseases, Centre International de Recherche<br>Raad, Mathieu; Fondation Mérieux, Direction des Opérations Internationales<br>Rasoanandrasana, Saida; Hôpital Befelatanana, Service de Biologie<br>Raherinandrasana, Antso Hasina; Faculté de Médecine de l'Université d'Antananarivo, Département de Santé Publique<br>Andriananja, Volatiana; Hôpital Befelatanana, Service des Maladies Infectieuses<br>Raberahona, Mihaja; University Hospital Joseph Raseta Befelatanana, Infectious Diseases<br>Moore, Catrin; Saint George's Hospital, Centre for Neonatal and Paediatric Infection<br>Randria, Mamy; University of Antananarivo Faculty of Medicine; University Hospital Joseph Raseta Befelatanana, Infectious Diseases<br>Raskine, Laurent; Fondation Mérieux, Direction des Opérations Internationales<br>Vanhems, Philippe; Hospices Civils de Lyon, Service d'Hygiène, Epidémiologie et Prévention, Lyon, France; Université Claude Bernard Lyon 1, 2. Public Health, Epidemiology & Evolutionary Ecology of Infectious Diseases (PHE3ID) team, Centre International de Recherche en Infectiologie (CIRI), Inserm U1111, CNRS UMR5308<br>Babin, François-Xavier; Fondation Mérieux, Direction des Opérations Internationales
**Primary Subject Heading**:	Infectious diseases
Secondary Subject Heading:	Epidemiology, Diagnostics
Keywords:	BACTERIOLOGY, Protocols & guidelines < HEALTH SERVICES ADMINISTRATION & MANAGEMENT, Diagnostic microbiology < INFECTIOUS DISEASES, Epidemiology < INFECTIOUS DISEASES

SCHOLARONE™
Manuscripts

Implementation of an antibiotic resistance surveillance tool in Madagascar, the TSARA project: a prospective, observational, multicenter, hospital-based study protocol.

C.Elias[1,2], M. Raad[3], S. Rasoanandrasana[4], AH. Raherinandrasana[5,], V. Andriananja[6], M. Raberahona[6], C.E. Moore[7], M. Randria[4], L. Raskine[3], P Vanhems[1,2], FX. Babin[3]

1. Service Hygiène et Epidémiologie, Hospices Civils de Lyon, Lyon, France
2. Public Health, Epidemiology & Evolutionary Ecology of Infectious Diseases (PHE3ID) team, Centre International de Recherche en Infectiologie (CIRI), Inserm U1111, CNRS UMR5308, ENS de Lyon, Université Claude Bernard Lyon 1, Lyon, France
3. Direction des Opérations Internationales, Fondation Mérieux, Lyon, France
4. Service de Biologie, Hôpital Befelatanana, Antananarivo, Madagascar
5. Département de Santé Publique, Faculté de Médecine de l'Université d'Antananarivo, Madagascar
6. Service des Maladies Infectieuses, Hôpital Befelatanana, Antananarivo, Madagascar
7. Centre for Neonatal and Paediatric Infection, St. George's, University of London, London

Corresponding Author:

Dr Christelle ELIAS

Service Hygiène et Epidémiologie, Hospices Civils de Lyon, Lyon

165 Chemin du Grand Revoyet, 69645 PIERRE-BENITE Cedex

christelle.elias@chu-lyon.fr

Phone: +33 4 78 86 12 73

Keywords: Antimicrobial Resistance, AMR, Antimicrobial Stewardship, Developing countries, Guidelines, Hospital, Low- and- middle-income countries, Madagascar, Surveillance

For peer review only - http://bmjopen.bmj.com/site/about/guidelines.xhtml

Abstract

Introduction: Antimicrobial resistance (AMR) has become a significant public health threat. Without any interventions, it has been modelled that AMR will account for an estimated 10 million deaths annually by 2050, this mainly affects low- and- middle-income countries. AMR has a systemic negative perspective affecting the overall healthcare system down to the patient's personal outcome. In response to this issue, the WHO urged countries to provide antimicrobial stewardship programs (ASP). ASP in hospitals are a vital component of national action plans for AMR, and have been shown to significantly reduce AMR, in particular in low-income countries such as Madagascar.

As part of an ASP, AMR surveillance provides essential information needed to guide medical practice. We developed an AMR surveillance tool - TSARA - with the support of the Mérieux Foundation. TSARA combines bacteriological and clinical information to provide a better understanding of the scope and the effects of AMR in Madagascar, where no such surveillance tool exists.

Methods and analysis: A prospective, observational, hospital-based study was carried out for data collection using a standardized data collection tool, called TSARA deployed in 2023 in 10 hospitals in Madagascar participating to the national Malagasy laboratory network (RESAMAD). Any hospitalized patient where the clinician decided to take a bacterial sample is included. As a prospective study, individual isolate-level data and antimicrobial susceptibility information on pathogens were collected routinely from the bacteriology laboratory and compiled with clinical information retrieved from face-to-face interviews with the patient and completed using medical records where necessary. Analysis of the local ecology, resistance rates and antibiotic prescription patterns were collected.

Ethics and dissemination: This protocol obtained ethical approval from the Malagasy Ethical Committee n°07-MSANP/SG/AGMED/CNPV/CERBM on January 24, 2023. Findings generated were shared to national health stakeholders, microbiologists, members of the RESAMAD Network and the Malagasy academic society of Infectious diseases.

For peer review only - http://bmjopen.bmj.com/site/about/guidelines.xhtml

Strengths and limitations of this study

- TSARA is a prospective, observational and multicentre protocol capturing data from hospitalized patients.
- TSARA is an AMR surveillance tool that compiles microbiological and clinical data, including information on antibiotic prescriptions implemented in Malagasy hospitals belonging to the RESAMAD network.
- The data collection is performed on an electronic tablet which limits data entry bias.
- The TSARA project is highly dependent on the infrastructure and the available resources in Malagasy laboratories.

For peer review only

For peer review only - http://bmjopen.bmj.com/site/about/guidelines.xhtml

Introduction

Antimicrobial resistance (AMR) is a global health threat associated with a poor prognosis and an increased mortality in the population infected with resistant bacteria. In the coming decades, deaths attributable to AMR will overcome the mortality due to non-communicable diseases through the lack of treatment options (1). Recent data from the Global Burden of Disease (2) suggested that the impact of AMR on health is even more important than previous estimations (1). To address the increasing problem, the World Health Organization (WHO) published the Global Action Plan on AMR in 2015 (3) and urged countries to build national action plans aiming to prioritise interventions to combat AMR rapidly. One of the objectives was to implement and strengthen surveillance systems to monitor AMR as a first step of an antimicrobial stewardship program (ASP) (4), in particular in hospital settings where high levels of resistance occur. In addition to these measures, the WHO also advocates for the implementation of antibiotic management policies adapted to local resistance patterns in hospitals (5–8). Setting up an ASP is challenging, especially in low- and- middle-income countries (LMICs) due to the lack of infrastructure, good quality data and governance (7,9). For a country like Madagascar, where robust healthcare facilities, infrastructures and laboratories are very scarce, collecting resistance data remains a challenge.

Madagascar is the fifth largest island in the world, but is considered a low-income country according to the World Bank (10). Health coverage in Madagascar is extremely limited and the patient has to directly pay for the majority of health care (i.e. diagnostics, treatment, consumables, surgery). Most drugs - especially antibiotics - are dispensed only with a medical prescription. However, in Madagascar, it is very common to deliver such medicines "Over The Counter" and antibiotics can be sold easily outside hospital settings, for those who can afford them, without any medical prescription. Lack or substandard infection control practices contribute significantly to the spread of bacterial resistance, both in the community (through AMR/antibiotics in wastewater) and in hospitals (hospital hygiene and nosocomial transmission). The culture of systematic treatment against an infectious agent encourages the misuse and the overconsumption of antibiotics, prescribed or not. At the hospital level, AMR has a greater effect, as patients are in hospital for several days, receive more care, have invasive procedures performed and thus are more at risk of transmission of bacteria and healthcare-associated infections. Appropriate hygiene measures and standard precautions are difficult to implement in low-resources settings, where there is for instance an average of five hospital beds per room in a Malagasy university hospital.

For peer review only - http://bmjopen.bmj.com/site/about/guidelines.xhtml

Outside of the RESAMAD laboratories, there are very few laboratories than can perform microbiology cultures and antimicrobial susceptibility testing (AST) in Madagascar and the burdened cost remains prohibitive for many patients. Targeted antibiotic therapy is therefore rarely prescribed and antibiotic treatment often remains empirical without any de-escalation. In this context of low access to microbiology laboratory combined with high rates of bacterial resistance at the national level, practitioners use broad-spectrum therapies, increasing the selection pressure on the bacteria and creating emerging resistances. Knowledge of the local resistance patterns is essential to adapt the clinical decision-making and have locally adapted recommendations (8). Table 1 provides a SWOT (Strengths, Weaknesses, Opportunities and Threats) analysis as an overview of what is currently in place and the starting points of the implementation of an AMR surveillance tool in Madagascar.

Table 1. SWOT analysis of the implementation of an AMR surveillance tool in hospitals in Madagascar

**Strengths**	**Weaknesses**
– Laboratory network RESAMAD (11) currently in place, already transferring data to the WHO through the GLASS-AMR module<br>– Active support of international stakeholders<br>– Identification of highly motivated Malagasy practitioners willing to work on AMR and ASP<br>– Existence of several scientific societies (infectious diseases, pediatrics, medical biology[1])	– Little knowledge on the local epidemiology of AMR<br>– Collection of antibiograms not performed on a daily basis<br>– Lack of visibility on antibiotic prescriptions in hospitals and their efficiency<br>– Implementation only doable where good laboratories exist<br>– Bacteriology results sometimes delivered to the prescriber in a paper format
**Opportunities**	**Threats**
– Capture high level antibiotic use in hospitals<br>– Existing antibiotic recommendations in place, but they are not adapted to local resistance patterns (12)<br>– Existing AMR protocols including the surveillance of antimicrobial use	– Challenging implementation<br>– Substantial missing data<br>– Personnel require training<br>– Lack of funding<br>– Political instability<br>– Climate disorders

[1] Société de Pathologie Infectieuse de Madagascar, Société Malgache de Pédiatrie, SOMABIO

For peer review only - http://bmjopen.bmj.com/site/about/guidelines.xhtml

In Madagascar as well as in multiple developing countries, the Mérieux Foundation has been strengthening clinical laboratories in the fight against infectious diseases for decades to enhance the quality and access to clinical laboratories aiming at preventing infections and improving patient care (13). The Mérieux Foundation has been investing on the fight against AMR supporting countries to develop infrastructures, laboratories, personnel training and building a laboratory network to strengthen laboratory-based surveillance of infectious diseases. As a result, in 2022, 14 Malagasy laboratories were in the RESAMAD (Réseau des Laboratoires à Madagascar) laboratory network, they were all able to perform bacteriology diagnosis, this was created in 2007 and has risen to 27 laboratories across the country to date (RESAMAD - Fondation Mérieux (fondation-merieux.org)).

Given this context, and the challenge to combat AMR in healthcare settings, ASP in hospitals are a vital component of national action plans for AMR, and have been shown to significantly reduce AMR, in particular in low-income country such as Madagascar (9,14). As part of an ASP, AMR surveillance provides essential information needed to guide medical practice. An AMR surveillance tool called TSARA (Technique de Surveillance Actualisée de la Résistance aux Antimicrobiens) was developed thanks to the support of the Mérieux Foundation, with the objective of combining patient data with laboratory and epidemiological surveillance data to provide a better understanding of the scope and the effects of AMR in Madagascar, where no such surveillance tool was available, in order to treat patients more effectively. It will allow monitoring and follow-up compared to the scarce existing prescribing data (15). The TSARA project also aligns with the antimicrobial surveillance program led by the Direction of epidemiological surveillance and Response of the Ministry of Health in the context of One Health and in collaboration with other Ministries to tackle AMR in Madagascar. TSARA also answers one of the objective of the Malagasy national action plan on AMR (16).

For peer review only - http://bmjopen.bmj.com/site/about/guidelines.xhtml

Objectives

One of the most important benefits of AMR surveillance at the pathogen level is to achieve a standardized, comparable, and easily validated data collection method for quality data that is used to implement better patient treatment and hospital/national guidelines (17). The data allows an optimization of the patient care by adapting treatment guidelines according the patient demographics and type of infection, taking into consideration of the circulating pathogens and local resistance patterns. The primary objective is:

1. To institute a systematic approach towards microbiological data and clinical data collection and analysis of antimicrobial susceptibility information to optimize antimicrobial therapy in Malagasy hospitals.

The secondary objectives are:

1. To establish timely reporting of information on pathogens of interest and their antimicrobial susceptibility profiles to medical doctors;
2. To allow monitoring of the hospital antibiotic prescription (quantity, quality and spectrum, concordance with bacteriological results);
3. To provide data to the laboratory quality assessment and identify areas for improvement.

For peer review only

Methods and analysis

Study design and setting

Study design

TSARA is a prospective, observational, multicenter, hospital-based study based on a standardized data collection tool deployed in ten facilities with both adult and pediatric patients in Madagascar (Figure 1), collecting microbiological, demographic, prescription and clinical data in one place.

Figure 1. Map of the laboratories and implementation sites of TSARA in Madagascar

Outcome measures

The outcomes of interest will be categorized into behavioural, clinical and microbiological outcomes. Behavioural outcomes include changes in prescribing practices among prescribers and compliance to AMS protocols. In the TSARA project, we consider an adapted antibiotic prescription as a behavioral outcome, defined as:

1. A prescription of an antibiotic therapy consistent with the national antibiotic guidelines, or
2. A prescription of an antibiotic therapy considering the laboratory results including antibiotic susceptibility testing results.

Then, a metric will be used to describe the number and the proportion of the adapted antibiotic prescriptions.

Microbiological outcomes include a description of the bacteria isolated by culture, bacterial resistance rates as well as the delay between the sample being collected and the laboratory results communicated to the prescriber (in days and hours, Supplementary material 1 and 2).

Clinical outcomes include mortality defined as death occurring during the patient's hospital stay or the length of stay defined as the duration between the admission date and discharge date from the hospital (Supplementary material 1 and 3).

Setting and laboratory capacity

For peer review only - http://bmjopen.bmj.com/site/about/guidelines.xhtml

All laboratories participating in the TSARA project are part of the nationwide RESAMAD network in Madagascar (11). Description of the infrastructure in each hospital included in the TSARA project is presented in Supplementary material 4.

These laboratories perform diagnostics and antimicrobial susceptibility testing for infectious diseases, and have the capacity to perform phenotypic confirmation of the presence of resistance. To participate in TSARA, laboratories have to meet minimal quality standards requirements defined by the RESAMAD network and participate in an external quality assessment program. The laboratories also have the required infrastructure, equipment, supplies and resources to perform AST following EUCAST standards. The reporting of results to clinicians are made by qualified and trained technicians supervised by clinical microbiologists. The Mérieux Foundation performs regular training to laboratory staff and support the purchase of consumables (i.e. Petri dish, antibiotic disks) and reagents.

Study population

The population of the study includes all patients (adults and children) arriving at the hospital who are inpatients and have bacterial samples taken by the physician and sent to the laboratory.

Inclusion criteria

All patients arriving at each hospital, who have a bacterial culture performed are included in the study. All positive and negative bacteriological samples from hospitalized patients in one of the participating institutions from January 2023 onwards will be included.

Exclusion criteria

The following criteria will be excluded from the TSARA project:

- All non-bacteriological specimens (virological, fungal, parasitic)
- All samples from patients not hospitalized >24 hours (emergency, consultation, outpatient hospitalization)
- All environmental samples (water, air, surfaces, medical devices)
- All post-mortem samples
- All samples reported as contaminated defined as results that do not contribute to clinical decision-making

For peer review only - http://bmjopen.bmj.com/site/about/guidelines.xhtml

- All specimens for which culture was not possible
- All specimens with laboratory non-compliance
- All the patients or legal guardians who object to be included in the TSARA project
- All patients with severe cognitive impairment
- All patients who do not have a full understanding of the French or Malagasy language

Participant timeline

Enrolled patients will be followed up during their hospital stay until discharge or death. Information on the vital status (alive, deceased, discharged or transferred to another hospital) will be collected.

Patient and public involvement

Patients and the public were not involved in the design of this study.

Data collection

Individual isolate-level data and AST on pathogens were collected routinely from the bacteriology laboratory using a first questionnaire (Supplementary material 1). These data were compiled using a second questionnaire with basic clinical, demographic, and epidemiological information (including current antibiotic prescription) retrieved daily through face-to-face interviews with the patient and completed by medical records where necessary (Supplementary material 2). A third questionnaire was then filled in regarding follow-up data (Supplementary material 3). The data retrieved from TSARA meet the criteria and are linked to the GRAM project that seeks to estimate the burden of AMR worldwide (17, 18).

To enhance data collection and limit data entry bias, data were directly captured on a tablet connected to the Epicollect5® platform (https://five.epicollect.net). Data were collected by Malagasy investigators (medical interns, biologists or clinical research associates) using tablets dedicated to this project, each of whom received specific training on the TSARA methodology prior to the start of the study. The investigator will examine the bacteriological samples daily, or as frequently as possible. A new eligible sample corresponds to a new inclusion in the TSARA project.

For peer review only - http://bmjopen.bmj.com/site/about/guidelines.xhtml

Statistical methods

Sample size

TSARA is a surveillance project, as such there is no calculation on the number of subjects needed *a priori*. Statistical analyses will be adapted *a posteriori* according to the total number of inclusions during the project period and the power needed to obtain valid results.

Description of the statistical methods

Any anomalies in the data will be checked with medical records during data cleaning. Data will be validated by quality control to identify outliers and inconsistencies by means of consistency tests (e.g. filling in the inclusion criteria, date of admission to hospital later than the date of admission to the department, date of death later than the date of onset of symptoms).

Descriptive summary tables with numbers and frequencies will be drawn up. Similarly, visual representations of the variables may be made to detect irregular or missing values. Any changes to the data will be documented and recorded separately from the raw database. Recoded variables will also be filled in (e.g. age categories).

A flow chart will be developed to describe the number of eligible, included and excluded patients and samples in our study. A descriptive analysis of the entire baseline population will be undertaken to identify the baseline characteristics of the study population (the specimen, bacterial, clinical and patient levels).

Categorical variables will be reported as frequencies and percentages of the total population. Quantitative variables will be described using the mean and standard deviation if the distribution is normal, or the median and interquartile range if the distribution does not follow the normal distribution. The range with the minimum and maximum values will be filled in. For each variable, the proportion of missing values will be indicated.

Stratified analyses will also be provided to present results by facility, department, type of sample, and bacteria of interest (including species, and antibiotic resistance of interest) as well as by type of antibiotics prescribed. In addition, given the fact that data are collected for the adult and pediatric population, a stratified analysis will be performed for both populations.

A two-group comparison using univariate and then multivariate analysis and a logistic regression will be performed to estimate the associations between an adapted antibiotic therapy and the other variables. The Odds-Ratio and p-value of each test will be reported. Only the variables with a p-value

For peer review only - http://bmjopen.bmj.com/site/about/guidelines.xhtml

lower than 0.2 will be retained to perform the multivariate analyses. In addition to the presentation of the multivariate analyses, the most efficient multivariate model will be presented after a top-down selection of only those variables with a p-value less than 0.05. Stratified analyses will be conducted to identify potential confounders (comparison of crude and adjusted Odds-Ratios) and interaction factors (Wald's test of homogeneity).

These analyses will be performed using the Chi-squared test or Fisher's exact test for qualitative variables, and the Student's t test or the Mann-Whitney test (depending on the nature of the variable or the size of the sample) for quantitative variables. A significance level of less than 0.05 is considered associated.

Patient survival will be calculated from date of enrolment until in-hospital death, based on the Kaplan–Meier method. Follow-up will be censored at 14 days and 28 days. Survival distributions will be compared by the log-rank test. Variables independently associated with survival will be identified with a Cox regression model based on relative hazard with a 95% confidence interval.

Missing data and outliers

Missing data will be documented individually. In the case of multiple missing data or if there is evidence of bias in the missing data on the variables of interest, multiple imputation methods may be used at the study level to substitute missing values. Imputation will be performed if the proportion of missing data does not exceed 20%. If this is the case, the variable will be removed from the statistical analysis.

A sensitivity analysis will be performed to compare the raw database (including missing data) with the imputed data.

Outliers were be prevented by a blocking input mask, alert messages and an input guide. However, if new outliers are discovered after the quality checks and during the analyses, they will be corrected as missing data. The scripts allowing these corrections will be communicated.

Data management and archiving

Case report form

The case report form only includes data necessary for participation in the TSARA project. All information required by the protocol is recorded in the anonymized case report forms. Data are

For peer review only - http://bmjopen.bmj.com/site/about/guidelines.xhtml

collected as they are obtained in the laboratory and then at the patient's bedside, and recorded in these case report forms explicitly.

These case report forms are set up in each of the facilities using the electronic tablets equipped with Epicollect applications, including the TSARA project questionnaires. An instruction book to help the investigators use this tool was provided. The investigators are responsible for the accuracy, quality and relevance of all data entered, under the responsibility of the lead investigator.

These case report forms are associated with de-anonymized forms, in paper format, containing the first and last names of the patients enrolled, as well as an identification number shared between the paper and online forms. These second de-anonymized forms stay within the hospital and should allow possible corrections or deletions of data upon request of the patients enrolled.

Data management

Collected data are computerized by the epidemiologist in charge of the project. Electronic databases are anonymous and locked with a password known only by the scientific staff. These data will be kept for a minimum of 15 years after the end of the study.

Archiving

The sponsor will keep the study documents (protocol and annexes, possible amendments, information forms, case report form, statistical analysis plan and output and the final study report) for a minimum of 15 years. After this period, the sponsor will be consulted before any data are destroyed. Study-related documents and reports may be subject to audit or inspection by the sponsor and/or other authorized bodies. No relocation or destruction will be made without the consent of the sponsor. At the end of the 15 years, the sponsor will be consulted for destruction. All data, documents and reports will be subject to audit or inspection.

<u>Confidentiality</u>

All personal data on the study participants will remain strictly confidential. To respect their privacy and for confidentiality, all participant details will be anonymous for the purpose of database preparation. Study subjects are coded with 4 numerical numbers.

For peer review only - http://bmjopen.bmj.com/site/about/guidelines.xhtml

Ethics and dissemination

Ethics

The TSARA study was approved by the National Ethics Committee in Madagascar on 24 January 2023 (n°07-MSANP/SG/AGMED/CNPV/CERBM).

Informed consent

Patients are fully and fairly informed, in understandable terms, of the objectives, their rights to refuse to participate in the study, or the opportunity to withdraw at any time.

All of this information is included on an information form given to the patient, and the patient's agreement will be collected by the investigator being included and will be recorded in the patient's source file.

The information will be communicated orally in French or in Malagasy by the investigators. An information form is also available in paper format in French and in Malagasy. When conditions permit, if the eligible patient does not speak French or Malagasy, the investigators will seek to volunteer a third party to provide a translation (a relative of the patient, or available hospital personnel).

Regulatory compliance

The study will be conducted in accordance with applicable laws and regulations currently in place in Madagascar.

Withdrawal criteria

Subjects may request to withdraw from the study at any time, without explanation, for any reason. In the event of premature exit, the investigator should document the reasons as completely as possible.

Stopping the research study

The Mérieux Foundation reserves the right to discontinue the study at any time, if it is determined that the inclusion objectives are not met, or if the protocol is not respected in one or more participating institutions, or subject to other circumstances beyond the control of the Mérieux Foundation (e.g.

For peer review only

For peer review only - http://bmjopen.bmj.com/site/about/guidelines.xhtml

political instability). In case of premature termination of the study in one of the institutions, the information will be transmitted by the sponsor to the Malagasy interlocutors.

Protocol amendments

In the eventuality of changes in the existing protocol that significantly affect the scope or the scientific quality of the investigation, an amendment containing a verbatim description of the changes and reference (date and number) to the submission that contained the original protocol will be submitted to the ethical committee for their approval.

Dissemination

Results and scientific reports that emerge from this study will be made publically available. This is under the responsibility of the principal investigator in agreement with the associated investigators. Results will be reported following the guidelines from the STrengthening the Reporting of OBservational studies in Epidemiology consortium (www.strobe-statement.org) and STROBE-AMS (19). Publication rules will follow international recommendations. The findings will also be shared with national health and sport authorities.

Authorship will follow the guidelines established by the International Committee of Medical Journal Editors (https://www.icmje.org), which require substantive contributions to the design, conduct, and interpretation and reporting of an epidemiological study.

These data will help the Malagasy scientific societies of infectious diseases (SPIM), pediatrics (SMP) and microbiology (SOMABIO) to tailor antibiotic recommendations according to the resistance patterns as presented in the Figure 2.

Figure 2: Data flow between the different stakeholders

For peer review only - http://bmjopen.bmj.com/site/about/guidelines.xhtml

For peer review only

Discussion

The TSARA project will provide original results that could 1) allow a more precise epidemiological knowledge of bacterial infections, prescribed antibiotic therapies and local resistance levels ; 2) Inspire, correct and tailor local practices, including recommendations and guidelines for empiric antibiotic therapy according to local resistance patterns ; 3) encourage communication and use of AST results with and between clinicians ; 4) allow monitoring of hospital antibiotic prescription (quantity, quality and spectrum, concordance with bacteriological results) and 5) provide evidence to improve laboratory services and interactions with clinicians.

Surveillance is the primary strategy for tracking emerging drug resistance in the population, and thus provides a tool to pick up any unusual organisms early and perform appropriate action. Regular dissemination of AMR data is key to elaborate local, evidence-based recommendations and revise the AMR national policy.

<u>Shortcomings</u>

Implementing TSARA as an AMR laboratory and patient-based surveillance system presents some challenges. First, all the patients included in the study were hospitalized, and are self-paying for the majority of healthcare costs which induces a selection bias and may hamper the representativeness of the data. Mainly tertiary care hospitals belonging to the RESAMAD laboratory network were included due to their laboratory capacities, secondary and primary care centers were under-represented in TSARA. Consequently, results from the AMR data generated might be inflated as these facilities harbour more vulnerable and sick patients, who may have previously taken antibiotics and been in hospital and therefore be subject to higher antibiotic exposure. Additionally, clinicians may prescribe antibiotics in the absence of microbiological documentation or for the treatment of non-bacterial infections, even though such practices could contribute to elevated rates of antibiotic resistance. This tool, although innovative, is highly dependent on the capacity and the willingness of staff, on laboratory capacity, which can sometimes be heterogeneous within the hospitals and over the time, depending on the turnover of the staff and the availability of consumables in country. The TSARA project can be implemented only in a facility where laboratory services are functional, robust and where bacterial cultures and AST are performed routinely. In addition, patient treatment could be influenced by a delay in obtaining the culture results. In Madagascar, as well as many LMICs, it is common to use diagnostic microbiology testing after treatment failure to improve on broad-spectrum antibiotics (20), which also overestimates local AMR rates. Negative perception of the laboratory

For peer review only - http://bmjopen.bmj.com/site/about/guidelines.xhtml

capacity and associated costs could explain the unwillingness to perform microbiology testing by the clinicians.

No clear standard definition of multidrug-resistant bacteria has been set up by Malagasy health authorities, which results in difficulties in the data interpretation and comparison. Data collection currently relies on paper-based data capture, where no electronic medical records currently exist in the facilities. The quality of data may be another obstacle to getting validated results and there might be exposed to intersite variability. Clinical data and follow up data could also be subject to a substantial proportion of missing data, as these are harder to retrieve than microbiological data. Lastly, the investigator may have a lack of motivation to collect the data as it could be perceived as added work with a limited incentive or reward.

For peer review only

Opportunities

The culture to collect of laboratory and clinical data is a serious challenge in LMICs. In 2021, the Mérieux Foundation implemented the Lab Book 3.0 (21), a Laboratory Information System (LIS) which helps to computerize clinical laboratory data, in all the RESAMAD laboratories. This has both improved data collection and ensures data quality.

Additionally, the use of information and technology through a digital tool could be the next step for TSARA. It would encourage a timely reporting of AMR and clinical data ensuring the correct patient treatment. Thus, it may incentivize scientific societies to adjust national antibiotic guidelines according to the local resistance patterns and hence support prescribers to make better, more informed clinical decisions and antibiotic prescription. Given the expansion of e-Health and connected devices, we imagine this tool to be integrated in a prescription assistance device on a mobile application as a future solution to combat AMR. Presenting the clinician with the antibiotic therapy recommendation associated with the WHO AWaRe classification has already shown its deployment in the fight against AMR (6).

Furthermore, starting from January 2022, a change in the patient payment has occurred where any patient admitted to a Malagasy hospital with a life-threatening disease has received all the initial hospital care at no cost. Leadership and financial commitment from local health authorities has been invaluable (i.e. Direction of epidemiological surveillance and Response of the Ministry of Health), and is key to the sustainability of the project and its expansion across the country.

Thanks to TSARA, significant progress should be made in AMR surveillance and antimicrobial stewardship in Madagascar. This project will also participate in supporting the implementation of the

For peer review only - http://bmjopen.bmj.com/site/about/guidelines.xhtml

national action plan on AMR in Madagascar. Assessing the impact and the efficiency of this project i.e. monitoring antibiotic prescriptions and checking their compliance with recommendations still need to be explored as clinicians' behaviors also drive AMR. Despite some challenges, future opportunities are encouraging for TSARA but rely on financial investment and strong leadership from health authorities to warrant the added value, the sustainability and the potential expansion of this project.

For peer review only

References

1. O'Neill J., Davies S., Rex J., White L.J., Murray R. Review on Antimicrobial Resistance, Tackling Drug-Resistant Infections Globally: Final Report and Recommendations. London, UK: Wellcome Trust and UK Government; 2016.
2. Murray CJ, Ikuta KS, Sharara F, Swetschinski L, Robles Aguilar G, Gray A, et al. Global burden of bacterial antimicrobial resistance in 2019: a systematic analysis. The Lancet. 2022 Jan;S0140673621027240.
3. WHO Global Action Plan for AMR. World Health Organization; 2015.
4. Pulcini C, Binda F, Lamkang AS, Trett A, Charani E, Goff DA, et al. Developing core elements and checklist items for global hospital antimicrobial stewardship programmes: a consensus approach. Clin Microbiol Infect. 2019 Jan;25(1):20–5.
5. Tebano G, Li G, Beovic B, Bielicki J, Brink A, Enani MA, et al. Essential and forgotten antibiotics: An inventory in low- and middle-income countries. Int J Antimicrob Agents. 2019 Sep;54(3):273–82.
6. Budd E, Cramp E, Sharland M, Hand K, Howard P, Wilson P, et al. Adaptation of the WHO Essential Medicines List for national antibiotic stewardship policy in England: being AWaRe. J Antimicrob Chemother. 2019 Nov 1;74(11):3384–9.
7. Mendelson M, Morris AM, Thursky K, Pulcini C. How to start an antimicrobial stewardship programme in a hospital. Clin Microbiol Infect [Internet]. 2019 Aug [cited 2023 Jul 3]; Available from: https://linkinghub.elsevier.com/retrieve/pii/S1198743X19304483
8. Elias C, Moja L, Mertz D, Loeb M, Forte G, Magrini N. Guideline recommendations and antimicrobial resistance: the need for a change. BMJ Open. 2017 Jul;7(7):e016264.
9. Cox JA, Vlieghe E, Mendelson M, Wertheim H, Ndegwa L, Villegas MV, et al. Antibiotic stewardship in low- and middle-income countries: the same but different? Clin Microbiol Infect. 2017 Nov;23(11):812–8.
10. World Bank Country Classification [Internet]. [cited 2023 Jul 3]. Available from: https://datahelpdesk.worldbank.org/knowledgebase/articles/906519-world-bank-country-and-lending-groups
11. RESAMAD [Internet]. [cited 2023 Jul 3]. Available from: https://www.fondation-merieux.org/projets/resamad/
12. Randriatsarafara FM, Ralamboson J, Rakotoarivelo R, Raherinandrasana A, Andrianasolo R. Consommation d'antibiotiques au Centre Hospitalier Universitaire d'Antananarivo : prévalence et défis stratégiques. Santé Publique. 2015;27(2):249.

For peer review only - http://bmjopen.bmj.com/site/about/guidelines.xhtml

13. Fondation Mérieux [Internet]. [cited 2023 Jul 3]. Available from: https://www.fondation-merieux.org/en/who-we-are/

14. Morency-Potvin P, Schwartz DN, Weinstein RA. Antimicrobial Stewardship: How the Microbiology Laboratory Can Right the Ship. Clin Microbiol Rev. 2017 Jan;30(1):381–407.

15. Protocol for Enhanced Isolate-Level Antimicrobial Resistance Surveillance in the Americas. Primary Phase: Bloodstream Infections [Internet]. Pan American Health Organization; 2021 [cited 2022 Feb 2]. Available from: https://iris.paho.org/handle/10665.2/53275

16. Plan d'action national pour combattre la résistance aux antimicrobiens 2019-2023 [cited 2023 Dec 22] Available from: https://cdn.who.int/media/docs/default-source/antimicrobial-resistance/amr-spc-npm/nap-library/madagascar_2019_2023.pdf?sfvrsn=cf290d1c_1&download=true

17. GRAM protocol [Internet]. [cited 2023 Jul 3] https://www.tropicalmedicine.ox.ac.uk/gram/research/global-burden-of-bacterial-antimicrobial-resistance/

18. And Schnall J, Rajkhowa A, Ikuta K, Rao P, Moore CE. Surveillance and monitoring of antimicrobial resistance: limitations and lessons from the GRAM project. BMC Med [Internet]. déc 2019 [cited 2023 Jul 3];17(1). Available from: https://bmcmedicine.biomedcentral.com/articles/10.1186/s12916-019-1412-8

19. Tacconelli E, Cataldo MA, Paul M, et al. STROBE-AMS: recommendations to optimise reporting of epidemiological studies on antimicrobial resistance and informing improvement in antimicrobial stewardship. BMJ Open 2016;6:e010134. doi:10.1136/bmjopen-2015- 010134

20. Krockow EM, Colman AM, Chattoe-Brown E, Jenkins DR, Perera N, Mehtar S, et al. Balancing the risks to individual and society: a systematic review and synthesis of qualitative research on antibiotic prescribing behaviour in hospitals. J Hosp Infect. 2019 Apr;101(4):428–39.

21. Lab Book [Internet]. [cited 2023 Jul 3]. Available from: https://www.lab-book.org/

For peer review only

For peer review only - http://bmjopen.bmj.com/site/about/guidelines.xhtml

Authors' contributions

C. Elias, M. Raad, FX. Babin, L. Raskine, M. Raberahona, S. Rasoanandrasana, C. E. Moore and M. Randria designed the study.

C. Elias and M. Raad drafted the manuscript.

C.Elias, S. Rasoanandrasana, AH. Raherinandrasana, V. Andriananja, and L Raskine participated in the acquisition of data.

C.Elias, M. Raad, S. Rasoanandrasana, AH. Raherinandrasana, V. Andriananja, M. Raberahona, C.E. Moore M. Randria, L. Raskine, P Vanhems and FX. Babin have revised and approved the final version of the manuscript.

Funding

The TSARA project has received funding from:

- Fondation Mérieux
- PFIZER ISID Research Grant n°63019455

Competing interests

None declared.

Acknowledgements

The authors thank all the biologists, namely Dr Christian RAFALIMANANA, Dr Lalaina RAHAJAMANANA, Dr Zakasoa RAVAOARISAINA, Dr Tiana Andry RAZAFINIKASA, Dr Lalaina RAOELINA, Dr Ainamalala Catherine RAZAFINDRAKOTO, Dr Solotiana Rivo RAKOTOMALALA, Dr Jocia FENOMANANA, Dr Emile RAVELOMANDRANTO, Dr Irène RAKOTONIAINA, and the local investigators of the RESAMAD network for their participation in the TSARA project.

The authors are very thankful for Luciana RAKOTOARISOA and Johannie RAKOTONIAINA for their support in the implementation of the project locally.

For peer review only

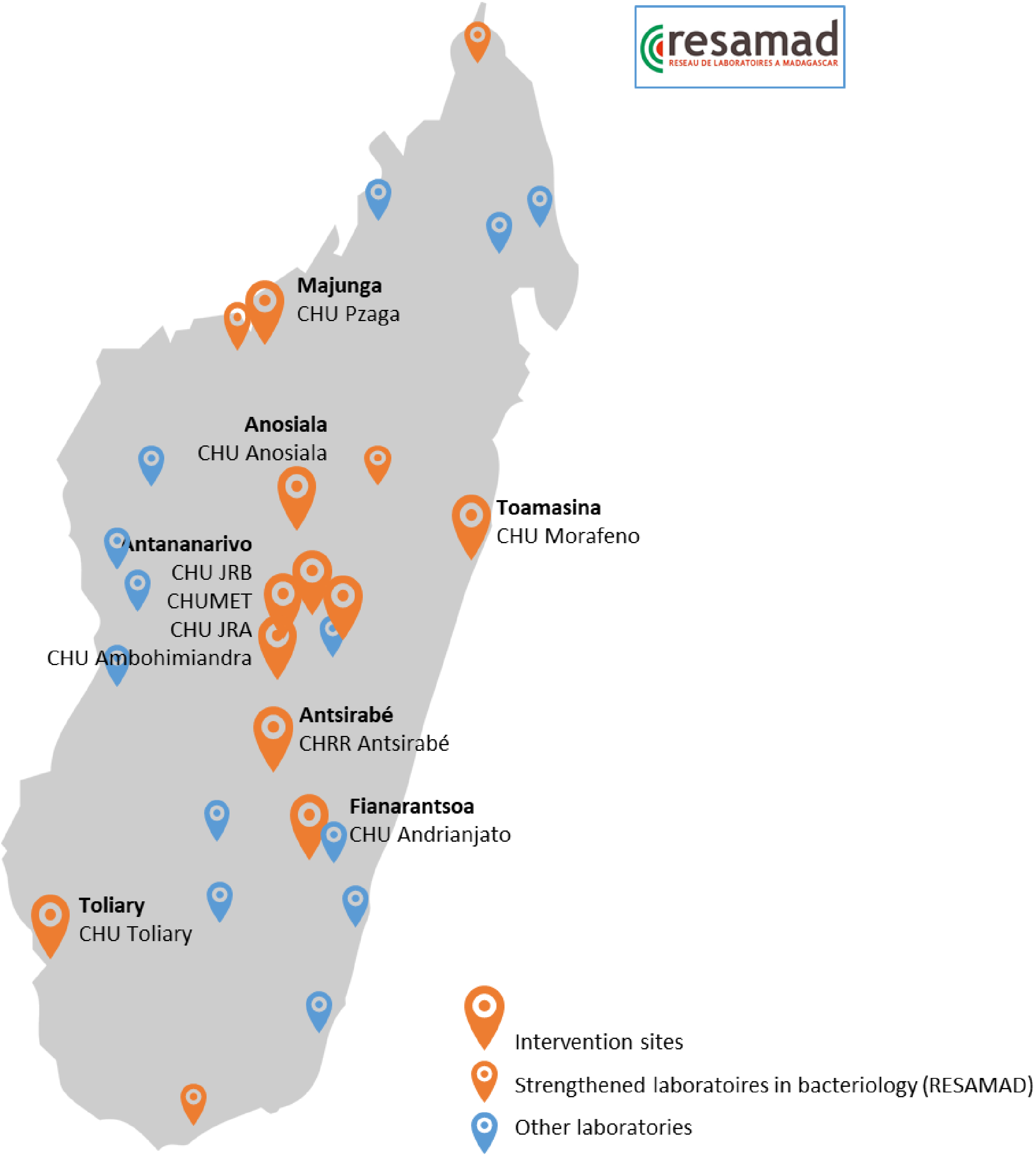
resamad
RESEAU DE LABORATOIRES A MADAGASCAR
Majunga
CHU Pzaga
Anosiala
CHU Anosiala
Toamasina
CHU Morafeno
Antananarivo
CHU JRB
CHUMET
CHU JRA
CHU Ambohimiandra
Antsirabé
CHRR Antsirabé
Fianarantsoa
CHU Andrianjato
Toliary
CHU Toliary
Intervention sites
Strengthened laboratoires in bacteriology (RESAMAD)
Other laboratories

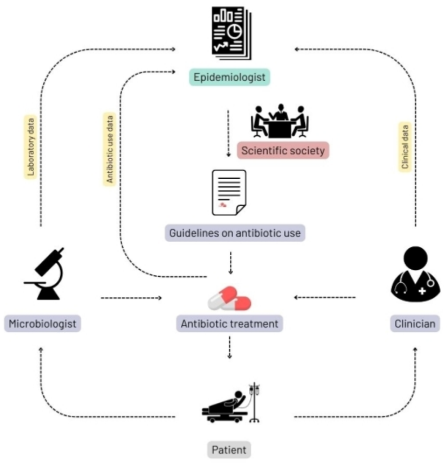

Figure 2. Data flow between the different stakeholders

162x91mm (300 x 300 DPI)

Supplementary material 1. TSARA microbiological questionnaire

Question	Name of the variable	Type of data	Format	Description
**Hospital where the sample was taken**	id_hosp	Single choice list	CHUJRB, CHUJRA, CHUMET, CHRR ANTSIRABE, CHU ANOSIALA, CHU MORAFENO , CHU PZAGA , CHU FIANARANTSOA, CHRR AMBOHIMIANDRA, CHU TULEAR	Hospital where the bacteriological sample was taken (hospital of inclusion)
**Patient identification number**	id_pat	Numeric	_ _ _ _ _ _ _ _ _ _	Patient's inclusion number
**Date of collection**	dt_pvt	Date	DD/MM/YYYY HH:MM	Date of collection
**Date of receipt of the sample in the laboratory**	dt_rec_pvt	Date	DD/MM/YYYY HH:MM	Date of receipt of the sample in the laboratory
**Date of bacteriological CR**	dt_cr_pvt	Date	DD/MM/YYYY HH:MM	Date of delivery of the result to the patient or prescribing physician
**Type of sampling**	pvt_type	Single choice list	Blood culture, Intravascular device, Ascites fluid, Cerebrospinal fluid,<br>Joint fluid, Respiratory specimen, Deep tissue specimen,<br>Genital specimen, Urine, Neonatal specimen and placenta, Superficial pus, Deep tissue specimen, Stool culture (not screening), Other specimen, Unknown	Type of sampling performed
**Positive culture**	culture	Single choice list	Yes/No	Identification of a microorganism on the culture
**If yes, identified pathogen**	bact	Single choice list	List of bacteria	Identification of the micro-organism on the sample
**Nalidixic acid**	nalidixic_acid	Single choice list	S, I, R, NOT TESTED	Sensitivity or resistance of the antibiotic tested
**Fusidic acid**	fusidic_acid	Single choice list	S, I, R, NOT TESTED	Sensitivity or resistance of the antibiotic tested
**Amikacin**	amikacin	Single choice list	S, I, R, NOT TESTED	Sensitivity or resistance of the antibiotic tested
**Amoxicillin**	amoxicillin	Single choice list	S, I, R, NOT TESTED	Sensitivity or resistance of the antibiotic tested
**Amoxicillin-clavulanic acid**	amoxicillin_clav	Single choice list	S, I, R, NOT TESTED	Sensitivity or resistance of the antibiotic tested
**Ampicillin**	ampicillin	Single choice list	S, I, R, NOT TESTED	Sensitivity or resistance of the antibiotic tested
**Ampicillin-Sulbactam**	ampicillin_sulbactam	Single choice list	S, I, R, NOT TESTED	Sensitivity or resistance of the antibiotic tested
**Azithromycin**	azithromycin	Single choice list	S, I, R, NOT TESTED	Sensitivity or resistance of the antibiotic tested
**Aztreonam**	aztreonam	Single choice list	S, I, R, NOT TESTED	Sensitivity or resistance of the antibiotic tested

For peer review only

**Cefadroxil**	cefadroxil	Single choice list	S, I, R, NOT TESTED	Sensitivity or resistance of the antibiotic tested
**Cefalexin**	cefalexin	Single choice list	S, I, R, NOT TESTED	Sensitivity or resistance of the antibiotic tested
**Cefalotin**	cefalotin	Single choice list	S, I, R, NOT TESTED	Sensitivity or resistance of the antibiotic tested
**Cefamandole**	cefamandole	Single choice list	S, I, R, NOT TESTED	Sensitivity or resistance of the antibiotic tested
**Cefazolin**	cefazolin	Single choice list	S, I, R, NOT TESTED	Sensitivity or resistance of the antibiotic tested
**Cefepime**	cefpime	Single choice list	S, I, R, NOT TESTED	Sensitivity or resistance of the antibiotic tested
**Cefixime**	cefixime	Single choice list	S, I, R, NOT TESTED	Sensitivity or resistance of the antibiotic tested
**Cefotaxime**	cefotaxime	Single choice list	S, I, R, NOT TESTED	Sensitivity or resistance of the antibiotic tested
**Cefoxitin**	cefoxitin	Single choice list	S, I, R, NOT TESTED	Sensitivity or resistance of the antibiotic tested
**Cefpodoxime**	cefpodoxime	Single choice list	S, I, R, NOT TESTED	Sensitivity or resistance of the antibiotic tested
**Ceftazidime**	ceftazidime	Single choice list	S, I, R, NOT TESTED	Sensitivity or resistance of the antibiotic tested
**Ceftriaxone**	ceftriaxone	Single choice list	S, I, R, NOT TESTED	Sensitivity or resistance of the antibiotic tested
**Cefuroxime**	cefuroxime	Single choice list	S, I, R, NOT TESTED	Sensitivity or resistance of the antibiotic tested
**Chloramphenicol**	chloramphenicol	Single choice list	S, I, R, NOT TESTED	Sensitivity or resistance of the antibiotic tested
**Chlortetracycline**	chlortetracycline	Single choice list	S, I, R, NOT TESTED	Sensitivity or resistance of the antibiotic tested
**Ciprofloxacin**	ciprofloxacin	Single choice list	S, I, R, NOT TESTED	Sensitivity or resistance of the antibiotic tested
**Clarithromycin**	clarithromycin	Single choice list	S, I, R, NOT TESTED	Sensitivity or resistance of the antibiotic tested
**Clindamycin**	clindamycin	Single choice list	S, I, R, NOT TESTED	Sensitivity or resistance of the antibiotic tested
**Cloxacillin**	cloxacillin	Single choice list	S, I, R, NOT TESTED	Sensitivity or resistance of the antibiotic tested
**Colistin**	colistin	Single choice list	S, I, R, NOT TESTED	Sensitivity or resistance of the antibiotic tested
**Daptomycin**	daptomycin	Single choice list	S, I, R, NOT TESTED	Sensitivity or resistance of the antibiotic tested
**Doxycycline**	doxycycline	Single choice list	S, I, R, NOT TESTED	Sensitivity or resistance of the antibiotic tested
**Ertapeneme**	ertapeneme	Single choice list	S, I, R, NOT TESTED	Sensitivity or resistance of the antibiotic tested
**Erythromycin**	erythromycin	Single choice list	S, I, R, NOT TESTED	Sensitivity or resistance of the antibiotic tested
**Fosfomycin**	fosfomycin	Single choice list	S, I, R, NOT TESTED	Sensitivity or resistance of the antibiotic tested
**Gentamicin**	gentamicin	Single choice list	S, I, R, NOT TESTED	Sensitivity or resistance of the antibiotic tested
**Imipeneme**	imipeneme	Single choice list	S, I, R, NOT TESTED	Sensitivity or resistance of the antibiotic tested
**Josamycin**	josamycin	Single choice list	S, I, R, NOT TESTED	Sensitivity or resistance of the antibiotic tested
**Levofloxacin**	levofloxacin	Single choice list	S, I, R, NOT TESTED	Sensitivity or resistance of the antibiotic tested

For peer review only

**Lincomycin**	lincomycin	Single choice list	S, I, R, NOT TESTED	Sensitivity or resistance of the antibiotic tested
**Linezolid**	linezolide	Single choice list	S, I, R, NOT TESTED	Sensitivity or resistance of the antibiotic tested
**Meropeneme**	meropeneme	Single choice list	S, I, R, NOT TESTED	Sensitivity or resistance of the antibiotic tested
**Metronidazole**	metronidazole	Single choice list	S, I, R, NOT TESTED	Sensitivity or resistance of the antibiotic tested
**Midecamycin**	midecamycin	Single choice list	S, I, R, NOT TESTED	Sensitivity or resistance of the antibiotic tested
**Minocycline**	minocycline	Single choice list	S, I, R, NOT TESTED	Sensitivity or resistance of the antibiotic tested
**Moxifloxacin**	moxifloxacin	Single choice list	S, I, R, NOT TESTED	Sensitivity or resistance of the antibiotic tested
**Neomycin**	neomycin	Single choice list	S, I, R, NOT TESTED	Sensitivity or resistance of the antibiotic tested
**Netilmycin**	netilmycin	Single choice list	S, I, R, NOT TESTED	Sensitivity or resistance of the antibiotic tested
**Nifuroxazide**	nifuroxazide	Single choice list	S, I, R, NOT TESTED	Sensitivity or resistance of the antibiotic tested
**Nitrofurantoin**	nitrofurantoin	Single choice list	S, I, R, NOT TESTED	Sensitivity or resistance of the antibiotic tested
**Norfloxacin**	norfloxacin	Single choice list	S, I, R, NOT TESTED	Sensitivity or resistance of the antibiotic tested
**Ofloxacin**	ofloxacin	Single choice list	S, I, R, NOT TESTED	Sensitivity or resistance of the antibiotic tested
**Oxacillin**	oxacillin	Single choice list	S, I, R, NOT TESTED	Sensitivity or resistance of the antibiotic tested
**Pefloxacin**	pefloxacin	Single choice list	S, I, R, NOT TESTED	Sensitivity or resistance of the antibiotic tested
**Piperacillin**	piperacillin	Single choice list	S, I, R, NOT TESTED	Sensitivity or resistance of the antibiotic tested
**Piperacillin-Tazobactam**	piperacillin_tazo	Single choice list	S, I, R, NOT TESTED	Sensitivity or resistance of the antibiotic tested
**Pivmecillinam**	pivmecillinam	Single choice list	S, I, R, NOT TESTED	Sensitivity or resistance of the antibiotic tested
**Polymyxin**	polymyxin	Single choice list	S, I, R, NOT TESTED	Sensitivity or resistance of the antibiotic tested
**Pristinamycin**	pristinamycin	Single choice list	S, I, R, NOT TESTED	Sensitivity or resistance of the antibiotic tested
**Rifampicin**	rifampin	Single choice list	S, I, R, NOT TESTED	Sensitivity or resistance of the antibiotic tested
**Spectinomycin**	spectinomycin	Single choice list	S, I, R, NOT TESTED	Sensitivity or resistance of the antibiotic tested
**Streptomycin**	streptomycin	Single choice list	S, I, R, NOT TESTED	Sensitivity or resistance of the antibiotic tested
**Sulfamethoxazole**	sulfamethoxazole	Single choice list	S, I, R, NOT TESTED	Sensitivity or resistance of the antibiotic tested
**Tedizolide**	tedizolide	Single choice list	S, I, R, NOT TESTED	Sensitivity or resistance of the antibiotic tested
**Teicoplanin**	teicoplanin	Single choice list	S, I, R, NOT TESTED	Sensitivity or resistance of the antibiotic tested
**Telithromycin**	telithromycin	Single choice list	S, I, R, NOT TESTED	Sensitivity or resistance of the antibiotic tested
**Temocillin**	temocillin	Single choice list	S, I, R, NOT TESTED	Sensitivity or resistance of the antibiotic tested
**Ticarcillin**	ticarcillin	Single choice list	S, I, R, NOT TESTED	Sensitivity or resistance of the antibiotic tested

For peer review only

**Ticarcillin-clavulanic acid**	ticarcillin_clav	Single choice list	S, I, R, NOT TESTED	Sensitivity or resistance of the antibiotic tested
**Tigecycline**	tigecycline	Single choice list	S, I, R, NOT TESTED	Sensitivity or resistance of the antibiotic tested
**Tobramycin**	tobramycin	Single choice list	S, I, R, NOT TESTED	Sensitivity or resistance of the antibiotic tested
**Vancomycin**	vancomycin	Single choice list	S, I, R, NOT TESTED	Sensitivity or resistance of the antibiotic tested

For peer review only

Supplementary material 2. TSARA Clinical Questionnaire

Question	Name of the variable	Type of data	Format	Description
**Date of visit in the room**	dt_visit	Date	DD/MM/YYYY	Date of patient/clinician visit to clinical service
**Gender**	gender	Unique choice	Male, Female	Sex of the patient
**Date of birth**	dt_naiss	Date	DD/MM/YYYY	Patient's date of birth
**Age**	age	Numeric	_ _ _	Age of patient at hospital admission in years
**Size**	size	Numeric	_ _ _	Patient height (cm) at admission
**Weight**	weight	Numeric	_ _ _	Patient weight (kg) at admission
**Status at birth**	status_naiss	Single choice list	Premature, Born at term	Premature status at the birth of the child
**Patient's residence**	place_resid	Single choice list	Urban, Rural	Patient's primary place of residence on admission
**Patient's region of residence**	reg_resid	Text	_ _ _ _ _ _ _ _ _ _ _ _ _ _ _ _	Patient's primary area of residence on admission
**Date of admission to hospital**	dt_adm_hosp	Date	DD/MM/YYYY	Date of hospital entry (TSARA participant)
**Patient's origin**	from	Single choice list	Home, Transfer to anotherhospital, Unknown	Patient's origin before admission to hospital
**Diagnosis at admission**	diag_adm	Single choice list	Infectious, Non-infectious, Unknown	Patient diagnosis on admission to hospital
**Type of service**	type_service	Single choice list	Medicine, Surgery, Intensive Care, Gyneco-Obstetrics,Pediatrics, Other	Type of service where the sample was taken
**Respiratory rate (beats per minute)**	freq_resp	Numeric	_ _ _	Patient's respiratory rate on admission
**Heart rate (beats per minute)**	freq_card	Numeric	_ _ _	Patient's heart rate on admission
**Systolic blood pressure (mmHg)**	pa_sys	Numeric	_ _ _	Systolic blood pressure on admission
**Disorder of consciousness**	awareness	Single choice list	Yes/No	Presence of disturbed consciousness at admission
**Peripheral venous access**	vvp	Single choice list	Yes/No	Placement of a PVV between admission and collection
**Central venous access**	vvc	Single choice list	Yes/No	Placement of a CVC between admission and

For peer review only

				collection
**Urinary catheterization**	probe_uro	Single choice list	Yes/No	Placement of a urinary catheter between admission andsampling
**Oro-tracheal intubation**	intubation	Single choice list	Yes/No	Intubation performed between admission and collection
**Reason for collection**	motif_pvt	Multiple choice list	List of reason for samples	Reason for collection
**Date of onset of symptoms**	dt_sympt	Date	DD/MM/YYYY	Date of onset of the first symptoms for which the sample was taken
**Antibiotic history within 3 months prior to admission**	atcd_atb	Single choice list	Yes/No	Use of ATB within 3 months of hospital admission
**If yes, specify ATBs**	type_atcd_atb	Multiple choice list	List of antibiotics	If yes, specify the type of ATB
**Antibiotic therapy during the stay before sampling**	atb_hosp	Single choice list	Yes/No	Antibiotic treatment prescribed between admission to hospital and sampling
**If yes, specify ATBs**	type_atb_hosp	Multiple choice list	List of antibiotics	If yes, specify the type of ATB
**Antibiotic therapy after receiving the antibiogram**	atb_post_atbg	Single choice list	Yes/No	Antibiotic treatment prescribed after receiving the antibiogram
**If yes, specify ATBs**	type_atb_post_atbg	Multiple choice list	List of antibiotics	If yes, specify the type of ATB
**History of hospitalization in the 6 months preceding thehospitalization**	atcd_hosp	Single choice list	Yes/No	History of hospitalization in the 6 months preceding thehospitalization
**History of surgery in the 6 months preceding hospitalization**	atcd_chir	Single choice list	Yes/No	History of surgery in the 6 months preceding hospitalization
**Comorbidities**	comorbidities	Multiple choice list	List of comorbidities	Presence of clinical comorbidities at admission
**Other comorbidities**	other_comorbidity	Free text		Other comorbidities

For peer review only

Supplementary material 3. TSARA III follow-up questionnaire

Question	Name of the variable	Type of data	Format	Description
**Date of discharge from hospital**	dt_end_hosp	Date	DD/MM/YYYY	End date of hospital stay or date of death if the patient has deceased.
**Exit diagnosis**	diag_hosp	Single choice list	List of diagnosis	
**Patient status at discharge**	hosp_output	Single choice list	Return home, Deceased, Transfer to another hospital	Patient status at the end of the hospital stay

For peer review only

Supplementary material 4. Description of facilities included in the TSARA project

	CHU[a] Joseph Ravoahangy Andrianavalona (JRA)	CHU Joseph Raseta Befalatanana (JRB)	CHU Mère-Enfant Tsaralalana (MET)	CHU Ambohimiandra	CHU Anosiala	CHRR[b] Antsirabe	CHU Andrianjato	CHU Pzaga	CHU Morafeno	CHU Toliary
**City**	Antananarivo	Antananarivo	Antananarivo	Antananarivo	Anosiala	Antsirabe	Fianarantsoa	Mahajanga	Toamasina	Toliary
**Type of hospital[c]**	Tertiary	Tertiary	Tertiary	Tertiary	Tertiary	Secondary	Tertiary	Tertiary	Tertiary	Tertiary
**University/Regional hospital**	Teaching	Teaching	Teaching	Teaching	Teaching	Regional	Teaching	Teaching	Teaching	Teaching
**Hospital size (number of beds)**	595 beds	450 beds	200 beds	44 beds	180 beds	70 beds	120 beds	303 beds	120 beds	40 beds
**Hospital ownership**	Public	Public	Public	Public	Public	Public	Public	Public	Public	Public
**Type of population**	Adult	Adult	Pediatrics	Adult & Pediatrics	Adult	Adult	Adult	Adult	Adult	Adult
**Presence of ICU[d]**	Yes	Yes	Yes	Yes	Yes	Yes	Yes	Yes	Yes	Yes
**Strengthened bacteriology laboratory**	Yes	Yes	Yes	Yes	Yes	Yes	Yes	Yes	Yes	Yes
**Diagnostic AST[e] method**	Disk diffusion (EUCAST v. 2021)	Disk diffusion (EUCAST v. 2021)	Disk diffusion (EUCAST v. 2021)	Disk diffusion (EUCAST v. 2021)	Disk diffusion (EUCAST v. 2021)	Disk diffusion (EUCAST v. 2021)	Disk diffusion (EUCAST v. 2021)	Disk diffusion (EUCAST v. 2021)	Disk diffusion (EUCAST v. 2021)	Disk diffusion (EUCAST v. 2021)
**Local guidelines on the use of antibiotics in place**	None	None	None	None	None	None	None	None	None	None

[a]CHU : Centre hospitalier Universitaire (Teaching hospital)
[b]CHRR : Centre Hospitalier de Référence régionale (Regional hospital)
[c]https://www.who.int/publications/i/item/WHO-EMP-IAU-2018.01
[d]ICU: Intensive Care Unit
[e]AST: Antibiotic susceptibility testing

For peer review only
